# Antioxidants for the Treatment of Breast Cancer: Are We There Yet?

**DOI:** 10.3390/antiox10020205

**Published:** 2021-01-31

**Authors:** Carmen Griñan-Lison, Jose L. Blaya-Cánovas, Araceli López-Tejada, Marta Ávalos-Moreno, Alba Navarro-Ocón, Francisca E. Cara, Adrián González-González, Jose A. Lorente, Juan A. Marchal, Sergio Granados-Principal

**Affiliations:** 1 Centre for Biomedical Research (CIBM), Biopathology and Regenerative Medicine Institute (IBIMER), University of Granada, 18100 Granada, Spain; carmengl@ugr.es (C.G.-L.); jmarchal@ugr.es (J.A.M.); 2 Instituto de Investigación Biosanitaria Ibs.GRANADA, University Hospitals of Granada-University of Granada, 18100 Granada, Spain; 3 Excellence Research Unit “Modeling Nature” (MNat), University of Granada, 18100 Granada, Spain; 4 GENYO, Centre for Genomics and Oncological Research, Pfizer/University of Granada/Andalusian Regional Government, 18016 Granada, Spain; jose.blaya@genyo.es (J.L.B.-C.); araceli.lopez@genyo.es (A.L.-T.); marta.avamo@gmail.com (M.Á.-M.); alba_n_ocon@hotmail.com (A.N.-O.); francisca.cara@genyo.es (F.E.C.); adrikangus@gmail.com (A.G.-G.); jose.lorente@genyo.es (J.A.L.); 5 Department of Legal Medicine, School of Medicine, University of Granada, 18016 Granada, Spain; 6 Department of Human Anatomy and Embryology, School of Medicine, University of Granada, 18016 Granada, Spain; 7 Department of Biochemistry and Molecular Biology II, School of Pharmacy, University of Granada, 18011 Granada, Spain

**Keywords:** breast cancer, antioxidants, reactive oxygen species, cancer stem cells, clinical trials, adjuvant therapy, cancer prevention

## Abstract

Breast cancer is the most frequent cancer and the leading cause of cancer death in women. Oxidative stress and the generation of reactive oxygen species (ROS) have been related to cancer progression. Compared to their normal counterparts, tumor cells show higher ROS levels and tight regulation of REDOX homeostasis to maintain a low degree of oxidative stress. Traditionally antioxidants have been extensively investigated to counteract breast carcinogenesis and tumor progression as chemopreventive agents; however, there is growing evidence indicating their potential as adjuvants for the treatment of breast cancer. Aimed to elucidate whether antioxidants could be a reality in the management of breast cancer patients, this review focuses on the latest investigations regarding the ambivalent role of antioxidants in the development of breast cancer, with special attention to the results derived from clinical trials, as well as their potential use as plausible agents in combination therapy and their power to ameliorate the side effects attributed to standard therapeutics. Data retrieved herein suggest that antioxidants play an important role in breast cancer prevention and the improvement of therapeutic efficacy; nevertheless, appropriate patient stratification based on “redoxidomics” or tumor subtype is mandatory in order to define the dosage for future standardized and personalized treatments of patients.

## 1. Introduction

### 1.1. Breast Cancer, an Overview

Globally, breast cancer is the most common type of cancer and the main cause of death by this disease among women. There were an estimated ~2 million new cases and 0.6 million breast cancer deaths in 2018 [1]. Breast cancer is classified into four subtypes, defined by the expression of estrogen and progesterone hormones, and human epidermal growth factor receptor 2 (HER2) [2,3,4,5]:

- Luminal A is hormone-receptor-positive (estrogen-receptor- and/or progesterone-receptor-positive) and HER2-negative. Luminal A cancers are low-grade, tend to grow slowly and have the best prognosis;
- Luminal B is hormone-receptor-positive (estrogen-receptor- and/or progesterone-receptor-positive) and either HER2-positive or HER2-negative. Luminal B cancers generally grow slightly faster than luminal A subtype;
- Triple-negative/basal-like is hormone-receptor-negative (estrogen-receptor- and progesterone-receptor-negative) and HER2-negative. This type of cancer is more common among younger and African American women. Most of these tumors have a high rate of brain and lung metastases.
- HER2-enriched is hormone-receptor-negative (estrogen-receptor- and progesterone-receptor-negative) and HER2-positive. HER2-enriched cancers can have a worse prognosis, but they are often successfully treated with targeted therapies aimed at the HER2 protein, such as trastuzumab.

Recent molecular and genetic studies have emphasized that breast cancer is a highly heterogeneous group of diseases, which differ in their prognosis and response to treatment. Certain risk factors, like genetic mutations, specifically BRCA1 and BRCA2 tumor suppressor genes [3,6], obesity (BMI ≥ 30 kg/m^2^) [3,7], exposure to X and γ radiation [3,7], early menarche, nulliparity, advanced age at first birth and advanced age of menopause [3,6], as well as alcohol consumption, smoking and diet, play an important role in the development of breast cancer [3,7].

Whereas breast cancer mortality rates have been declining in a number of highly developed countries, breast cancer incidence rates have increased, mainly due to changes in lifestyle over time. Early detection, improved and novel therapeutics, and more effective treatment regimens in breast cancer are currently on the focus of research [1,2,7,8].

### 1.2. Oxidative Stress and Reactive Oxygen Species in Cancer

Oxygen is a key requirement for any biological process so that the concentration thereof is controlled with great precision. However, a certain portion of the oxygen is partially reduced to reactive oxygen species (ROS), a large group of oxygen-derived small molecules, which include radicals and nonradical species, produced by various endogenous and exogenous substances in enzymatic and nonenzymatic reactions. Under excessive stress conditions, numerous ROS are generated, leading to an imbalance between free radicals and the antioxidant defense, also known as oxidative stress. If oxidative stress is too high for the cells to maintain their own homeostasis, it can cause cell death by triggering the activation of proapoptotic pathways, necrosis, autophagy and pro-angiogenic events, considered them all key-factors for the development of several disorders, including neurodegenerative, cardiovascular and cancer diseases [9,10,11].

Cancer cells are usually submitted to higher ROS levels that further stimulate a malignant phenotype, promoting sustained cell proliferation, cell survival, angiogenesis, metastasis and inflammation. Therefore, it is considered an established source of carcinogenesis [12,13]. In breast cancer, ROS and oxidative stress are involved in DNA damage, which can inhibit or induce transcription, signal transduction pathways, replication errors, genomic instability and activation of oncogenes. There are several risk factors in breast cancer associated with ROS-induction, such as aging, menopause, genetic predisposition or estrogens, which results in DNA damage and chromosomal aberrations, and hence, supporting the development and progression of the disease [11]. Provided that the regulation of oxidative stress and the maintenance of REDOX homeostasis are important factors in both tumor development and response to anticancer therapies, targeting REDOX regulation is emerging as a promising strategy for the treatment of breast cancer [13,14]. Glutathione (GSH) metabolism is essential for maintaining a correct REDOX balance, which protects tumor cells from stress and ensures their survival in solid malignancies. Therefore, combinations of GSH inhibitors with anticancer therapies may prove to be useful for killing cancer cells [14]. Likewise, alterations in mitochondrial biology have a crucial role in cell metabolism and homeostasis and are related to a progression in breast cancer, being ROS production one of the consequences associated with mitochondrial dysfunction. In this regard, differences in mitochondrial function among breast cancer subtypes drive distinct ROS production and dependence by tumor cells. For instance, mitochondria lead to enhanced ROS levels as reported in triple-negative breast cancer (TNBC), and therefore, targeting ROS and/or mitochondria could be a reasonable therapeutic approach in this breast cancer subtype [15].

### 1.3. Antioxidants

Antioxidants are compounds that inhibit the oxidation of other molecules. More specifically, attending to the mechanistic functions, they are agents that possess the ability to be hydrogen or electron donors. Humans have developed complex antioxidant systems, which work together with each other to protect cells against free-radical-derived damage and scavenge ROS by up-regulating antioxidant defenses, normalizing mitochondrial function, and blocking the process of oxidation by neutralization of free radicals [16]. Antioxidants can be natural, enzymatic and nonenzymatic, or synthetic compounds, e.g., enzymes like superoxide dismutase (SOD), catalase (CAT), or nutrient-delivered compounds that are consumed with the food as dietary supplements like vitamin C, tocopherols, tocotrienols, carotenoids, and vitamin E [17,18,19,20].

Since the middle of the 20th century, it has been demonstrated that antioxidants reduce the occurrence of different processes like aging, cancer, inflammation, or liver disease, among others. Antioxidants can be used as dietary supplements, prophylactic treatment of different diseases, or even to ameliorate the negative effects of chemo and radiotherapy by reducing the oxidative stress in cancer [17,18]. In addition, antioxidants may reduce the morbidity associated with the use of different chemotherapeutic drugs because of their major efficiency for decreasing chemotherapy-induced toxicity, which seems to increase survival rates in cancer patients and improve the therapeutic efficiency [12,21]. However, there is controversy about the efficacy of these compounds for the treatment of cancer and, more specifically, breast carcinogenesis, mainly because the same molecule may exhibit both antioxidant and pro-oxidant properties depending on the concentration, tumor cell type, timing and environmental conditions [22]. In this regard, it has been investigated the pro-oxidant state of pharmacological ascorbate (vitamin C) for the steady-state formation of ascorbate and H_2_O_2_ in the extracellular space and blood in rats through different routes of administration (i.v.; i.p. or oral) and those doses used in humans. It has been demonstrated that pharmacological ascorbate is a prodrug for the preferential steady-state formation of ascorbate and H_2_O_2_ in the extracellular space, but not in blood. Furthermore, an experiment in mice with a daily drug treatment regimen with ascorbate in tumors of pancreas, ovary and glioblastoma showed a very significant decrease in tumor growth, suggesting that pharmacological ascorbate is a pro-oxidant prodrug with therapeutic benefits in cancer [23,24]. In breast cancer, the antioxidant enzyme peroxiredoxin-1 (PRDX1) has been studied as a possible biomarker and therapeutic target because its downregulation significantly impaired the proliferation rate of breast cancer cells and tumor growth in an in vivo model of xenotransplanted PRDX1-deficient MCF-7 cells. Moreover, in TNBC, PRDX1 is crucial for maintaining the REDOX status and has a cytoprotective effect. Accordingly, PRDX1-knockdown resulted in the inhibition of cell growth in vitro and such effects were enhanced when it was combined with pro-oxidant agents like H_2_O_2_, glucose oxidase, sodium L-ascorbate or menadione [25,26].

For instance, when tumors are irradiated, endogenous antioxidants cause a reduction of free radicals induced by radiation therapy, which protects not only normal but also tumor cells, favoring their proliferation. Moreover, such a controversy becomes intensified because the doses of antioxidants that are administered in clinical trials are not well-defined and refined, and not all tumor types are dependent on ROS signaling; hence, no benefits from antioxidants should be expected [12]. Furthermore, it is important to note that antioxidants could show a lack of efficacy due to endogenous factors (e.g., genetic variability in enzymes), which would decrease their ability to reduce ROS levels, breakdown lipid membrane and, therefore, protect cells from oxidative stress. It is known that high ROS levels limit cancer cell survival on the onset of tumor initiation and progression. Hence, dietary supplementation with antioxidants at these stages could promote cancer cell survival and accelerate tumor growth. In this regard, intake of dietary antioxidants was found to worsen breast cancer prognosis in postmenopausal women, and enhance the rate of metastasis and decrease survival in mouse models of cancer [15,27,28,29,30]. Although clinical trials were not successful enough to demonstrate that antioxidants were effective as a potential monotherapy, these compounds are being slowly incorporated as either supplements or adjuvants in standard therapies [19,31,32]. In the present review, we address the role of different antioxidants that could potentially be used as therapeutic strategies in breast cancer. Specially, we focus on those current clinical investigations that study the use of antioxidants as plausible adjuvants in radiotherapy or chemotherapy for breast cancer patients and their potential to inhibit the side effects of different treatments.

## 2. Antioxidants and Breast Cancer

The effectiveness of natural products, including phenolics, flavonoids, carotenoids, etc., has shown to suppress early and late-stages of carcinogenesis, reflecting their ability to counteract certain upstream signals, such as genotoxic damage redox imbalances, and other forms of cellular stress. Epigenetic agents alone or in combination with conventional anticancer drugs may prove a significant advance [33]. There are many different dietary, synthetic and endogenous antioxidants that are being widely investigated. Nevertheless, we will focus herein on those with a high potential to be used as therapeutic agents for breast cancer in both preclinical and clinical studies, namely natural dietary antioxidants like melatonin, resveratrol, curcumin, vitamin E, vitamin C, vitamin D, carotenoids, hydroxytyrosol, epigallocatechin gallate, selenium and some synthetic antioxidants. We describe the clinical trials (registered or not in ClinicalTrials.gov) focused on antioxidant supplementation as plausible monotherapy or adjuvant therapy, as well as the most significant trials aimed to ameliorate therapy-derived side effects.

### 2.1. Melatonin

Melatonin (N-acetyl-5-methoxytryptamine) is an indoleamine mainly secreted by the pineal gland, which is related to circadian rhythms and regulates multiple physiological functions, e.g., the sleep/wake rhythm. Moreover, it is a well-known scavenger of excessive free radicals, which induces the synthesis of antioxidant enzymes, and is implicated in tumor growth inhibition, angiogenesis and metastasis in cancer [34]. Accordingly, low levels of melatonin might be a risk factor in breast cancer [35]. This molecule is also an anti-estrogenic agent that modulates estrogen pathways and decreases tumor growth [11,36]. Interestingly, women with altered production of melatonin, caused by a circadian disruption after exposure to dim light at night, have intrinsic resistance to paclitaxel via epigenetic mechanisms, which increase signal transducer and activator of transcription 3 (STAT3) expression, which is frequently overexpressed in paclitaxel-resistant breast cancer. Administration of melatonin could therefore reestablish sensitivity of breast tumors to paclitaxel and stimulate tumor regression [37]. Additionally, mammary tumor cells treated with melatonin showed decreased proliferation, and it was effective in controlling metastatic breast cancer, both in vitro and in vivo, by modulating Rho-associated kinase protein-1 (ROCK-1) inhibition [38]. Melatonin acts as a radioprotective agent because it is able to neutralize different types of free radicals produced by ionizing radiation and several pro-oxidant enzymes (NADPH oxidase, xanthine oxidase or nitric oxide synthase). Therefore, it is capable of radiosensitizing breast cancer cells, and more specifically, those positive for estrogen receptor (ER). Melatonin also exhibits neuroprotective abilities by counteracting the adverse effects of adjuvant chemotherapy on cognitive function, sleep quality and depressive symptoms. Hence, it could be a novel adjuvant therapy to improve the existing breast cancer treatments [39,40,41,42]. Furthermore, melatonin can increase the efficacy of chemotherapeutics in combinatory treatments [40]. Accordingly, normal levels of melatonin combined with Taxol have been shown to prevent breast cancer metastasis through the inhibition of the DJ-1/KLF17/ID-1-signaling pathway [43]. However, there are conflicting studies regarding urine melatonin levels and the risk of developing breast cancer in postmenopausal women [44,45], as well as studies discussing how much melatonin could protect against undesirable effects (such as altered gene expression and post-translational protein modifications) caused by chemotherapy or radiotherapy treatments. Moreover, supplementation with dietary melatonin in humans has a safety profile without toxic effects; De Seabra and colleagues studied the toxicity of melatonin (10 mg) during 28 days in volunteers versus a placebo. They did not observe differences or adverse effects in several plasma and urinary biochemical parameters, as well as on sleep-related behavioral aspects, between both groups [46]. However, possible side effects of melatonin have been related to fatigue, mood, sleep disorders or psychomotor and neurocognitive performance. Most side effects can be easily managed by dosing in accordance with natural circadian rhythms [47,48]. Several clinical trials with melatonin are currently in progress to elucidate the correct administration and possible side effects in patients with breast cancer (Table 1). However, a genetic profile and powerful analytical tools are needed to better understand the antitumor effects of melatonin, which may probably be a promising agent in the list of drugs for the treatment of breast cancer. We would like to highlight three clinical trials, emphasizing their common characteristics and differences. First of all, they are all randomized, double-blinded, placebo-controlled trials, which agree that the administration of melatonin results in an improvement in sleep quality and expected effect since regulation of sleep/wake rhythm is one of its qualities. Nevertheless, these trials do not coincide with dosage nor patients’ situation [41,49,50,51,52,53]. NCT01355523 included post-surgery patients with a dose of 6 mg of melatonin. Their study succeeds as a decrease of depressive symptoms were observed in the timeline of 3 months after the surgery [49]. In a second trial, Palmer et al. studied the neuroprotective effect of 20 mg of this compound in patients undergoing their first cycle of adjuvant chemotherapy (NCT03205033). Even when the underlying mechanism was unknown, different neurological tests concluded that melatonin ameliorated cognitive functions [41]. Furthermore, pain perception was evaluated following some tests, obtaining that 20 mg of melatonin reduced it in comparison to the placebo group. However, this effect was not a consequence of an improvement in sleep quality as there was no correlation between them [53]. Finally, a non-registered clinical trial on postmenopausal breast cancer survivors evaluated the effect of 3 mg of melatonin on breast cancer biomarkers such as estradiol and insulin-like growth factor I (IGF-1). No significant differences were obtained after a period of four months administering this dosage, whereas, in other studies with larger doses, these biomarkers were reduced. Additionally, an improvement in sleep disturbances was observed, as we previously pointed out, but no conclusive results were observed in depression symptoms or hot flashes [51,52]. To sum up, at the present time, clinical trials have only evaluated the side effects of melatonin during breast cancer treatment.

### 2.2. Resveratrol

Resveratrol (3,5,4’-trihydroxy-transstilbene) is a non-flavonoid polyphenol present in several foods, including grapes, peanuts, berries or beans. This compound has a radical-scavenging activity, which prevents the access of oxidizing agents to the lipids, acting as a chemopreventive and antitumor agent [54]. The antitumor activity of resveratrol is mediated through the inhibition of several cell-signaling pathways such as Hippo/YAP, which is implicated in tumorigeneses, tumor progression and invasion [55]. Resveratrol was demonstrated to decrease breast cancer cell proliferation by the regulation of p53 and ERα protein expression [56], cell cycle arrest and induction of apoptosis, being more cytotoxic in 4T1 TNBC cells [57]. It also caused the up-regulation of the ATP2A3 gene, which triggered apoptosis and changes in intracellular Ca^2+^ regulation in MCF-7 and MDA-MB-231 breast cancer cell lines [58]. Moreover, resveratrol-loaded solid lipid nanoparticles, designed to treat MDA-MB-231 cells, showed superior ability in inhibiting the cell proliferation in comparison to the administration of free resveratrol [59]. New analogs of resveratrol, like 4-(E)-{(4-hydroxyphenylimino)-methylbenzene-1,2-diol} (HPIMBD) and 4-(E)-{(p-tolylimino)-methylbenzene-1,2-diol} (TIMBD), were found as strong antioxidants against breast cancer and prevented the development of cell growth by scavenging cellular ROS production, reducing oxidative DNA damage, increasing mRNA and protein expression of SOD3 and quinone oxidoreductase 1, and activating Nrf-signaling pathway [60]. The antioxidant capacity of resveratrol, as well as its effects on breast cancer cells in both in vitro and in vivo models, has been widely reported. However, several studies suggest that daily intake of resveratrol and other polyphenols could stimulate tumor growth in ER+ breast cancer due to the key role of estrogens in cancer cell proliferation. Therefore, the study of interactions between estrogens and polyphenols with estrogen precursors, like steroid dehydroepiandrosterone (DHEA), active estrogens or catechol estrogens are necessary for future clinical trials design. Moreover, another important aspect to consider is the selection of adequate cell and animal models that will allow obtaining more conclusive results regarding the future use of resveratrol as monotherapy for breast cancer patients [61,62]. Resveratrol is usually well-tolerated and shows no toxicity or adverse effects; however, several studies reported toxicity due to oral resveratrol at high doses (1000 mg/kg/day) in rats, however, doses up to 750 mg/kg/day for 3 months were well-tolerated and non-toxic. Similarly, resveratrol at 200 (mg/kg)/day in rats and 600 mg/kg/day in dogs did not cause toxic effects. In humans, a small proportion of case subjects showed different toxic effects such as increased blood bilirubin or headache, most of them related to high doses of resveratrol. Therefore, the data show that most of the adverse events occurred at the highest doses and in sporadic cases. Nonetheless, more trials would be necessary to better define the toxicity of this antioxidant [63,64]. Nowadays, clinical trials with resveratrol cannot provide enough information about its effects on breast cancer. Zhu et al. conducted a study on 39 women at high risk of breast cancer, resulting in novel conclusions about the protective effect after administration of 5 or 50 mg of trans-resveratrol twice a day for 12 weeks. This double-blinded placebo-controlled study analyzed the methylation of four cancer-related genes: p16, RASSF-1α, APC, CCND2. In a previous in vitro study, trans-resveratrol was shown to reduce DNA methylation and PGE2 levels, which was directly related to a decrease of RASSF-1α methylation and, consequently, supported the chemopreventive effects of resveratrol [65]. Furthermore, NCT01370889 evaluated the administration of 1 mg daily for 12 weeks of resveratrol in order to study its effects on systemic sex steroid hormones of overweight and obese postmenopausal women. Although no significant differences were obtained in serum level of estradiol, estrone and testosterone, it was observed an increase of sex steroid hormone-binding globulin and urinary 2-hydroxyestrone. This led to favorable effects on estrogen metabolism, suggesting a beneficial impact on these breast cancer risk factors. However, one participant concluded the study with an asymptomatic grade 4 side effect (elevation of liver enzymes) and two out of the 40 subjects presented grade 3 skin rashes [66]. Lastly, the clinical trial NCT03482401 studied the effect of a combination of 37 different phenolic compounds that were administered orally to 19 women previous to breast cancer surgery. The aim was the development of a complete metabolic profile in malignant and normal tissue, detecting metabolites of these phenolic compounds that did not exert antiproliferative or estrogenic/antiestrogenic activities in MCF-7 breast cancer cells [67]. Like other authors, we insist on the relevance and need for further studies. In Table 2, we summarized all clinical trials of resveratrol registered in www.clinicaltrials.gov that are related to breast cancer.

### 2.3. Curcumin

Curcumin (diferuloylmethane), a polyphenol derived from turmeric (*Curcuma longa*), possesses several properties, including anti-inflammatory, antitumor, anti-oxidative and chemopreventive effects on breast cancer. The antitumor effect of curcumin is exerted through different molecular-signaling networks involved in proliferation, ER, HER2 pathways and the regulation of genes related to breast cancer metastasis like TGF-α, TGFβ1, SERPINE1, PGAP3, MAP3K1, MAPK1, vimentin, among others [68,69,70]. Experimental evidence has shown that curcumin also regulates breast cancer cell proliferation by the arrest of the cell cycle at G2/M and promotion of apoptosis, which may be associated with the decrease of CDC25 and CDC2, the phosphorylation of NFκB, PI3K/Akt/mTOR, MAPK and JAK/STAT, the increase of p21 protein levels, the induction of the mitochondrial apoptotic pathway [71,72], the modulation of the tumor microenvironment and cancer immunity through activation of natural killer cells [73], or even by miRNAs like miR-21 [70,74]. Furthermore, WZ35, an analog of curcumin, showed anti-cancer properties in breast cancer cells through the regulation of a novel ROS/YAP/JNK pathway, which is involved in the induction of mitochondrial dysfunction and apoptosis in breast cancer cells [75]. Additionally, the use of curcumin in combination with chemotherapeutic agents is also being studied. It has been shown that the combination of curcumin with different drugs, like somatostatin, has cytostatic activity by the induction of changes in the composition of fatty acid in the membrane of breast cancer cells [76]. Other reports demonstrated that the combined treatment of curcumin and paclitaxel resulted in a higher level of apoptosis in breast cancer cell lines compared with monotherapy [77]. Similarly, the combination of metformin and curcumin also exhibited greater effects against tumor proliferation and growth by reducing vascular endothelial growth factor (VEGF) expression and inducing apoptosis with no signs of toxicity [78]. Moreover, a synergistic action of curcumin and quercetin was found against TNBC cells by modulating tumor suppressor genes, especially in women with a BRCA1 mutation [79,80]. Although there are several studies of the benefit of curcumin in human health, there is a scarcity of specific studies about adverse effects and long-term toxicity in humans. Preclinically, some studies to determine the toxicity profile of curcumin in rodents found no toxic, mutagenic or fatal effects when it was administered at different doses [81,82,83]. Among the registered clinical trials that are currently being carried out with curcumin (Table 3), only one of them uses curcumin as monotherapy, while the majority of clinical trials use curcumin as a dietary supplement in combination with chemotherapy or radiotherapy, being the last aimed at reducing the dermatitis effect of radiation. In fact, dermatitis occurs in 90% of patients treated with radiotherapy, especially in patients with breast, neck, or head cancer. A randomized, double blind, placebo-controlled clinical trial (NCT01042938) was carried out in breast cancer patients who received a radiation dose of 42.6–50.4 Gy during 16–33 sessions, and 390 mg curcumin, 75 mg demethoxycurcumin, 12.5 mg bisdemethoxycurcumin, or placebo were administered to patients in doses of 84 pills every seven days. After the fifth radiotherapy session, each patient underwent a weekly skin test. The group treated with curcumin showed significantly lower radiation dermatitis severity (RDS) levels than those in the placebo group. However, curcumin was not effective in patients with the most severe dermatitis, redness, or other symptoms such as pain or diarrhea [84]. Furthermore, Ryan et al. carried out a multi-site, randomized, placebo-controlled, blinded study (NCT02556632) testing curcumin in a topical way. In this trial, patients who received 44–66 Gy during 22–36 radiotherapy sessions were divided into three groups: HPR Plus (an FDA-approved medical device recommended for atopic dermatitis and radiation dermatitis), curcumin gel 4% (PsoriaGold^®^), and placebo, which were packaged in an air pump bottle with 48 gr of gel and applied thrice daily. Skin tests were carried out several times, from baseline to 2 weeks post-radiotherapy. Both curcumin and HPR Plus gels significantly reduced some symptoms of radiation dermatitis, like redness or itchiness, but no RDS scores. However, in patients with more severe radiations skin reaction curcumin decreased RDS scores, while both curcumin and HPR plus gels alleviated pain levels [85]. Additionally, curcumin has also been tested in combination with chemotherapeutics in a single institution, open-label, phase I clinical trial. Docetaxel is a drug extensively used against breast cancer, but not all patients respond well to treatment. Accordingly, the combination of these chemotherapeutic agents with non-cytotoxic compounds like curcumin to enhance its effect has been proposed. Dose-limiting toxicity (DLT) of this combination has been studied in patients under treatment with docetaxel (100 mg/m^2^), six times every three weeks, and curcumin administered in capsules ranging from 500 to 8000 mg. Neutropenia, leucopenia, diarrhea and dermatological were the principal adverse effects, which led to establish the DLT at 6000 mg/day. It has been shown that the combination of docetaxel and curcumin reduced levels of the CA15.3 tumor marker and VEGF antiangiogenic marker, but it was independent of curcumin dose. Hence, it could not be determined whether the reduction in those markers level was due to docetaxel alone, or its combination with curcumin [86]. Further research is needed to confirm this effect.

### 2.4. Vitamin E

Vitamin E is a common antioxidant supplement that prevents DNA damage caused by free radicals in breast, colon and prostate cancers, cardiovascular diseases, cataracts, arthritis and certain neurological disorders. The major forms of vitamin E are tocopherols (α-, β-, γ-, or δ-tocopherol) and tocotrienols, a family of fat-soluble phenolic compounds. They regulate peroxidation reactions, control free-radical production within the body and have known anticancer properties that reduce the risk of human cancer [87]. In breast cancer, it has been demonstrated that low levels of vitamin E are associated with an increased risk of suffering from this disease. Interestingly, whereas γ-δ-tocopherol and tocotrienol have a much lower systemic bioavailability, they have shown stronger cancer-preventive activities, and for the treatment of estrogen-mediated breast cancer [88,89], however, α-tocopherol succinate enhances the anti-tumor activity of pterostilbene in breast cancer cells [90]. Oppositely, another study demonstrated that vitamin E significantly promoted MCF7 cell proliferation by reducing ROS production and p53 expression [91]. In breast cancer patients receiving radiotherapy, the use of pentoxifylline (PTX) and vitamin E improved radiation-induced fibrosis and radiotherapy toxicity [92]. While the combination of tocopherols with other compounds like methotrexate, an analog of folic acid, seemed to enhance the anticancer activity in α-tocopherol-treated TNBC with high-doses, reduced anticancer activity was reported with low-doses [93]. Doxorubicin and cyclophosphamide, two drugs that are commonly used to treat breast cancer and can cause premature ovarian failure and infertility, potentially increased their in vitro chemotherapeutic efficacy against breast cancer cells with the addition of tocopherol whilst decreasing cytotoxicity towards ovarian granulosa cells [94]. Additional approaches showed that polymeric micelles comprising α-tocopherol and heparin, loaded with docetaxel, had more toxicity against breast cancer cells than free docetaxel [95]. Regarding the toxicity profile of vitamin E, it has been found to be very low after oral administration. In fact, in vivo studies indicated that vitamin E is not mutagenic, carcinogenic, or teratogenic. In humans, a daily dose of 100–300 mg of vitamin E can be considered non-toxic. Even on double-blind studies involving a large number of subjects, high oral doses of vitamin E (3200 mg/day) did not produce adverse effects. Remarkably, in some patients with vitamin K deficiency, high doses of vitamin E could worsen blood coagulation; hence its supplementation should be contraindicated in these patients [96,97,98]. When searching for the current clinical trials of vitamin E in breast cancer on www.clinicaltrials.gov, we retrieved 11 trials that are described in Table 4. Similar to most antioxidants, several clinical trials have focused on the prevention of treatment-derived side effects. Jacobson et al. studied the combination of vitamin E with PTX to reduce radiation-induced fibrosis in a randomized clinical trial (NCT00583700). Patients were irradiated with a total dose of 46.8–50.4 Gy and treated with 400 mg of PTX three times each day and 400 IU of vitamin E or placebo every day for six months after radiation. A tissue compliance meter was used to measure fibrosis severity. It was shown that radiation-induced fibrosis was significantly lower in the group treated with vitamin E than in the placebo group [99]. Conversely, other studies have focused on the effect of vitamin E in the treatment of breast cancer rather than the reduction of side effects. In fact, the combination of tamoxifen with tocotrienol showed an enhanced effect on the inhibition of breast cancer cell growth in vitro. In another clinical trial, patients were treated with 200 mg of tocotrienol rich fraction (TRF) or placebo, and 20 mg of tamoxifen each day for five years in a double-blinded, placebo-controlled pilot trial (NCT01157026). TRF administration caused a significantly increment in vitamin E levels in blood of patients that correlated with an enhancement of survival of 60%. However, this improvement was not statistically significant, as well as results in liver function, which were not altered [100]. In summary, like other antioxidants, vitamin E appears to reduce the negative effects of radiotherapy, such as fibrosis, but it remains unclear whether it actually has a direct effect on breast cancer survival.

### 2.5. Vitamin C

Vitamin C or ascorbic acid is a water-soluble compound that scavenges free radicals and suppresses chain initiation. Historically, this compound has been well known because its deficiency could lead to a fatal disease called scurvy and could only be cured with the administration of vitamin C. This compound is a potent antioxidant that contributes to immune defense and is deficient in patients with advanced stages of cancer. These effects are due to its ability to modulate the REDOX status of cells and to induce epigenetic modifications. Moreover, in the last years, it has been demonstrated that the intravenous administration of pharmacological doses of vitamin C kills cancer cells without being toxic to their normal counterparts [28]. In breast cancer, a meta-analysis of results from prospective studies has suggested that the use of vitamin C as a supplement after a cancer diagnosis may reduce the risk of patient mortality [101]. Interestingly, the role of vitamin C in energy metabolism suggests that it functions as a pro-oxidant with selective toxicity against specific types of tumor cells and, more specifically, breast cancer cells. Moreover, vitamin C has been shown to induce high ROS levels and oxidation of glutathione. Accordingly, high-dose of intravenous vitamin C and intravenous GSH have been used as complementary and adjuvant medicines in colon and breast cancer [102]. This antioxidant is usually combined with other agents to evaluate their inhibitory effect in mammary cancer models. Hanikoglu et al. studied the combined effects of vitamin C and somatostatin in MCF7 and MDA-MB-231 breast cancer cell lines and found changes in the membrane fatty acid composition and altered signaling pathways like MAPK and EGFR [103]. Similarly, the combination of vitamin C and auranofin could be efficient against TNBC, as well as other cancers with similar REDOX properties and PTGR1 expression levels [104]. Mostafavi and colleagues showed that the combined treatment of methotrexate with vitamin C inhibited TNBC cell growth through Nuclear factor erythroid 2-related factor 2 (Nrf2), which may act as a sensor for electrophilic stress. Therefore, such a combination could regulate the intracellular antioxidant response and be used as an adjuvant treatment for those cancer patients with Nrf2 overexpression in their tumor tissue [105]. In vitro studies showed that vitamin C promoted apoptosis in breast cancer cells by increasing TRAIL expression. Moreover, it was shown that high dose intravenous ascorbic acid therapy affected the levels of C-reactive protein (CRP) and proinflammatory cytokines, reduced inflammation and stimulated the production and activation of immune cells. However, it is necessary to point out that although vitamin C is not toxic and it is safe in low doses, it has been observed that oral doses >2 g generate side effects in a dose-dependent manner, such as abdominal pain or diarrhea. A safe daily dose of vitamin C in healthy people would be less than 500 mg per day; however, in people with kidney problems, it can be harmful because they could form oxalate stones. Even though more toxicological assays are needed, the optimal intake depends on its bioavailability in food, on the health conditions of the subject [106]. Nevertheless, despite the above commented, it is not certain whether there is a clinically strong positive effect of vitamin C supplementation in cancer patients on the overall survival [107,108]. In a randomized, 5-month study carried out by Suhail et al. protective effects of supplementation of vitamin C and E were evaluated in breast cancer patients undergoing chemotherapy. They tested the activity of some antioxidant enzymes (SOD, CAT, glutathione-S-transferase and glutathione reductase), levels of malondialdehyde and GSH, and DNA damage in peripheral lymphocytes. These parameters were measured before administration of first chemotherapy and were compared versus healthy controls. Later, patients received chemotherapy alone or in combination with oral supplementation of 500 mg vitamin C and 400 mg vitamin E. Similar to non-treated patients, chemotherapy group significantly lower levels of antioxidant enzymes, more extensive lipid peroxidation and DNA damage versus healthy controls, whereas supplementation with vitamins C and E versus chemotherapy produced a significant increase in the levels of antioxidant enzymes, enhancement of GSH, reduced malondialdehyde and DNA damage. In other words, the addition of these vitamins restored antioxidant status and decrease DNA damage, which are two side effects of chemotherapy, supporting the hypothesis of their beneficial effect [109]. Nevertheless, the limited number of ongoing early phase clinical trials (Table 5) do not provide evidence enough to reveal the potential of vitamin C in the therapy of breast cancer. The design of adequate cancer models and appropriate clinical trials are necessary in order to further understand the mechanism of action of vitamin C, which would facilitate the determination of the correct dose and timing [110].

### 2.6. Vitamin D

Vitamin D comprises a variety of hydrophobic molecules, and its principal form is vitamin D_3_, also known as cholecalciferol [111]. The biotransformation in the organism of the different forms of vitamin D starts with 7-dehydrocholesterol, which is transformed in a non-enzymatic synthesis in the human skin, producing vitamin D_3_. This second form can also be obtained through the diet, but it is a minor source since not many natural products contain it. Cholecalciferol is then transformed in the liver resulting in 25-hydroxyvitamin D_3_ (25(OH)D_3_), also called calcidiol, whose serum concentration is the biomarker for vitamin D status. Lastly, calcidiol suffers another hydroxylation, which occurs principally in kidneys, obtaining 1α,25-dihydroxyvitamin D_3_ (1,25(OH)_2_D_3_) or calcitriol, the most biologically active form of vitamin D with a high affinity for the vitamin D receptor [111,112]. Vitamin D is well-known because of its role in calcium homeostasis and osteosynthesis, and its deficiency produces bone malformation such as rickets [112]. It also plays an antioxidant role, inducing the expression of several antioxidant enzymes. For instance, in the MCF-7 breast cancer cell line, calcitriol increases the expression of thioredoxin reductase 1 [113]. Moreover, this hydrophobic molecule also contributes to the correct function of the immune, muscular and nervous systems [111]. Upon binding to its nuclear receptor, the complex acts as a transcription factor and regulates the immunometabolism. Among its properties, vitamin D controls cellular proliferation, differentiation and apoptosis of immune cells, which grow at a fast rate [112]. With regard to cancer, low levels of vitamin D have been associated with an elevated risk of cancer incidence. However, in the case of breast cancer, we found controversial results that can be a consequence of the heterogeneity of the different subtypes of breast cancer [112]. In vitro assays with breast cancer cell lines provided an interesting insight, where treatment with 1,25(OH)_2_D_3_ was shown to induce anti-proliferative effects via inhibition of genes coding for cyclins and apoptosis through the stimulation of inhibitory signals and genes like BCL2 family proteins [112]. Furthermore, in another study, 1,25(OH)_2_D_3_ inhibited cancer stem cell renewal by the reduction of mammosphere forming ability in MCF10DCIS and SUM159 cell lines, as well as by the downregulation of the Notch-signaling pathway [114]. Similarly, in vivo experiments confirmed that vitamin D not only reduced tumor initiation and growth but also inhibited the Wnt/β-catenin pathway and the generation of cancer stem cells (CSCs) [115]. In recent years, the popularity of vitamin D has grown, and its use as a dietary supplement has been increasing without adequate medical supervision. When vitamin D intake is excessive (serum concentrations of 25-hydroxyvitamin D greater than 150 ng/mL), and for long periods, it produces several toxicity-related side effects that can result in exogenous hypervitaminosis D, with symptoms of hypercalcemia, confusion, apathy, abdominal pain, polyuria, polydipsia, and dehydration. Although vitamin D toxicity is rare, serious effects on human health can occur if not identified immediately. For instance, acute kidney injury has been reported in the Kashmir Valley of the Indian subcontinent due to an overdose and irrational use of vitamin D by individuals [116,117].

To date, several clinical trials have investigated the effect of vitamin D in breast cancer, and the most relevant ones are summarized in Table 6. It should be pointed out that there is controversy about the importance of the parameters chosen for analysis. Certainly, intake of vitamin D and serum concentration levels of 25(OH)D_3_ or 1,25(OH)_2_D_3_ is not the same and, therefore, conclusions may differ depending on the parameter used [112]. In a first evaluation of the VITAL study (NCT01169259), a randomized, double-blinded, placebo-controlled trial, with a two-by-two factorial design (2000 IU/day of vitamin D_3_ and 1 g/day of marine omega-3 fatty acids) with a total of 25,871 participants and a median follow-up of 5.3 years, supplementation with vitamin D_3_ was not associated with a lower risk of development of cancer and no adverse events were identified [118]. Nevertheless, in secondary analysis, significant differences were found due to the refinement of results. After excluding early follow-up data and dividing patients into subgroups depending on their body mass index, it was suggested that the intake of vitamin D_3_ reduced the incidence of advanced and/or mortality rates among the normal-weight population. Authors argued that the reason for this difference could be the decreased bioactivity of vitamin D in overweight or obese people, as well as the fact that using the same dose of vitamin D_3_ would result in a greater volumetric dilution and, consequently, a minor variation of active vitamin D levels. Unfortunately, case numbers for mortality due to breast cancer were too small to be interpreted [119]. This argument was supported by other authors like Grant et al., who defended the importance of designing protocols and analyses including serum 25(OH)D_3_ concentrations as the main analysis parameter rather than using the intake dose of vitamin D_3_ [120]. Additionally, the WHEL study (NCT00003787) assessed the association between vitamin D and recurrence in breast cancer survivors. In this prospective cohort study of 3085 breast cancer survivors, the amount of dietary, supplemental and total vitamin D was analyzed. No significant association was observed between serum 25(OH)D_3_ levels and breast cancer recurrence, nor when data were stratified by menopausal status. Surprisingly, there was a significant inverse association between dietary vitamin D intake and recurrence in premenopausal women, but the authors emphasized the necessity of being cautious in interpreting these results [121]. In a secondary evaluation, Jacobs et al. studied the correlation between 25(OH)D_3_ serum concentrations and stage of breast cancer, resulting in no significant association [122]. Furthermore, the effects of vitamin D in breast tumors have also been studied. NCT01948128 evaluated the intake of a high dose of vitamin D3 (40,000 IU/day) versus placebo for 2–6 weeks in relation to breast tumor proliferation and apoptosis previous to surgery. Even when 25(OH)D_3_ serum levels were three times higher than in the placebo group, no significant effects on tumor proliferation and apoptosis were observed [123]. Regardless of their aim, most clinical trials deal with the musculoskeletal symptoms that occur in breast cancer because of treatment with aromatase inhibitors. Interestingly, a previous study showed that most patients with arthralgia and myalgia had low serum levels of 25(OH)D_3_ (83.3 and 88% respectively) [124]. However, other studies did not show a correlation between the two groups [125]. Based on the possible relationship between vitamin D levels and arthralgia, several clinical trials have been conducted by supplementing patients on aromatase inhibitor therapy with vitamin D. All these trials include women with hormone receptor-positive breast cancer to be treated with an aromatase inhibitor and had low blood levels of vitamin D. Patients given with a supplement of 400 IU vitamin D once a week for 8 weeks, and once a month until the end of the study, showed an improvement in pain, especially those patients with lower vitamin D levels at the beginning of the study [126]. However, in another trial which studied the development of arthralgia in patients treated with high or low doses of vitamin D (50,000 IU oral vitamin D_3_ per week for 12 weeks, followed by 2000 IU daily for 40 weeks, or 800 IU vitamin D_3_ daily for 52 weeks, respectively) no significant differences were seen between the two groups, even though vitamin D levels in blood were higher in the high dose group [127]. Oppositely, Khan et al. (NCT00867217) showed that treatment with vitamin D (30,000 IU per week and 600 IU daily in a high-dose group, or 600 IU daily in a low-dose group) caused a decrease in arthralgia cases in that group with higher doses, although this difference was not significant [128]. The absence of a placebo group in both clinical trials did not allow to observe the effect of vitamin D supplementation at low concentrations in comparison to the absence of supplementation. In conclusion, the relationship between vitamin D blood levels and the development of arthralgia in women with breast cancer under aromatase inhibitor treatment remains unclear, as well as the potential use of vitamin D supplementation to reduce this side effect.

### 2.7. Carotenoids

Biological activities attributed to carotenoids include not only powerful antioxidant properties but also the inhibition of malignant tumor growth and proliferation, the induction of apoptosis, modulation of gene expression and immune response. These compounds can minimize the adverse effects of chemotherapeutic drugs on normal cells by acting as antioxidants without interfering with their cytotoxic effects on cancer cells [129]. As with other antioxidants, there is conflicting information regarding their effects on breast cancer, such as the saffron carotenoids crocin and crocetin, which showed a strong radical-scavenging activity and SOD inhibition in MCF-7 cells in vitro, but an enhanced activity in breast tumor-bearing BALB/c mice after one month of treatment [130]. Cancer chemoprevention by dietary carotenoids involves several mechanisms, including those related to tumorigenesis like growth factor-signaling, cell cycle progression, the Wnt/β-catenin pathway and inflammatory cytokines [131]. Studies in the plasma of breast cancer patients evaluated the association between plasmatic concentrations of carotenoids, retinol, tocopherol, and vitamin C and the risk of breast cancer, and found that higher concentrations of plasma β-carotene and α-carotene were related to lower breast cancer risk in HER2 tumors [132]. In addition, studies about changes in diet and lifestyle in breast cancer survivors, where plasma carotenoids and other antioxidants concentration were measured, showed, after a short-term diet and exercise intervention in overweight/obese breast cancer survivors, positive changes in fatty acid biomarkers, which could be relevant for a breast cancer prognosis [133,134]. When we focus on the toxicity of these compounds, carotenoids are generally non-toxic. Experimental studies in animals have shown that β-carotene is neither mutagenic nor teratogenic. However, there are some exceptions, especially with a large intake of high doses that produce hypercarotenemia or high serum concentrations of carotene, causing a reversible yellowish color of the skin. In addition, consumption of more than 20–30 mg per day of β-carotene for prolonged periods was associated with an increased risk of lung and stomach cancer in smokers [135,136]. The majority of trials are based on the premise that higher plasma carotenoid concentrations, obtained from specific diets that include foods rich in carotenoids (carrots, fruits, and other vegetables), are protective in relation to breast cancer recurrence. Table 7 summarizes registered clinical trials that are being conducted to validate the effect of carotenoids in breast cancer patients. Among them, the trial NCT00000611 measured serum concentrations of carotenoids, retinol and tocopherols in women (6%) within the Women’s Health Initiative Clinical Trials at baseline and at one, three and six years later, to evaluate the association with postmenopausal breast cancer risk, confirming the evidence of an inverse association of α-carotene and β-carotene with breast cancer [137]. In another clinical report, Butalla et al. showed similar results by analyzing oxidative stress (8-iso-PGF2α) and inflammation in breast cancer survivors with overweight after a daily intake of orange and carrot juice for three weeks. It was concluded that the consumption of carrot juice favored an increase in plasma total carotenoids what diminished oxidative stress, but no changes in inflammatory biomarkers in breast cancer survivors were found [138]. Similarly, Rock et al. examined the relationship between plasma carotenoid concentration and women with a history of early-stage breast cancer for five years. These authors observed that women in the highest quartile of total plasma carotenoid concentration had a significantly reduced risk of a new breast cancer event [139]. Contrarily, a similar trial concluded that there was not a protective association with dietary carotenoid intake in 207 postmenopausal breast cancer survivors. However, it was demonstrated a significant inverse association between total plasma carotenoid concentrations and oxidative stress by urinary 8-oxo-7,8-dihydro-2’-deoxyguanosine (8-OHdG or 8-oxodG) [140]. In summary, most clinical trials with carotenoids in breast cancer focus on lifestyle, diet and recurrence in breast cancer survivors. In fact, there is even a study about music therapy for breast cancer patients in which carotenoids concentration is measured (NCT04446624). Further clinical assays must be done to really validate any positive effect of carotenoids in breast cancer patients.

### 2.8. Hydroxytyrosol

Hydroxytyrosol (HT), a phenylethanoid, is a type of phenolic phytochemical with powerful antioxidant and anticancer properties. In nature, HT is found in olives, in the form of its oleanolic acid or ester oleuropein. HT has different effects in normoxic and hypoxic conditions, being particularly effective in a hypoxic environment by modulating the transcription and translation of members of the PGC-1α/ERRα and PGC-1α/Nrf2 pathways and downregulating the expression of BCL-2 and COX-2 proteins. For this reason, the hypoxic environment of tumor cells should be considered when analyzing this antioxidant [141,142,143]. Another effect of HT and oleuropein is the suppression of migration and invasion via activation of autophagy in ER-positive breast cancer cells and by blocking hepatocyte growth factor or 3-methyladenine, an inductor of cell migration [143]. In vivo studies of HT showed anti-cancer effects in mammary tumor-bearing rats, reducing tumor growth and cell proliferation. HT was related to several genes associated with these processes, as well as the Wnt-signaling pathway, which promoted a high expression of SFRP4 (Secreted Frizzled-Related Protein 4) [144]. In addition, researchers demonstrated the ability of HT to reduce the chemotherapy-associated cardiotoxicity that doxorubicin provoked in animal models of breast cancer [145]. It has been proposed that a combination of HT and paclitaxel would ensure a less oxidative impact of chemotherapeutic drugs, which could improve the health of breast cancer patients [146]. In fact, it was demonstrated that breast cancer patients receiving a supplement of HT (15 mg/day) in combination with epirubicin and cyclophosphamide, followed by taxanes, showed significantly lower levels of Tissue Inhibitor of Metalloproteinases-1 (TIMP-1) in plasma versus control group, what could evolve less likelihood of cell proliferation, apoptosis, and metastasis [147]. Regarding the toxicity of consumption of pure HT, it should be mentioned that there are few clinical studies in this respect, and most of them are carried out with extracts derived from olives or olive oil (≥70% of HT content). In vitro and in vivo toxicity studies (subchronic and acute), reviewed elsewhere, have shown that there was no mortality and morbidity by different administration methods, causing only piloerection and local redness in the inoculation or soft feces after the doses. Pure HT at 500 mg/kg/day for 13 days did not reveal adverse effects, or disturbances in organ function or their structure, in rats [148,149]. Among the clinical trials assessing the efficacy of this antioxidant (Table 8), a pilot study evaluated whether the combination of HT, omega-3 fatty acids, and curcumin would reduce CRP and musculoskeletal symptoms in breast cancer patients receiving adjuvant hormonal therapies. This prospective, multicenter, open-label, single arm, clinical trial enrolled postmenopausal breast cancer patients (n = 45) with elevated CRP and taking predominantly aromatase inhibitors, to receive a combination of HT, omega-3 fatty acids, and curcumin for 1 month. They observed a reduction in inflammation and pain associated with a reduction in CRP in breast cancer patients with aromatase-induced musculoskeletal symptoms (NCT01819948) [150]. However, in order to fully understand the effect of this phenol in breast cancer patients, we not only must wait for the release of results from the ongoing clinical trials, but also other studies are necessary to help elucidate the potential use of HT in breast cancer therapies.

### 2.9. Epigallocatechin Gallate

Epigallocatechin gallate (EGCG), the most abundant biological constituent of green tea, has suppressive effects on different types of cancer, including breast cancer, through the regulation of different signaling pathways. It was found to be interacting directly with Pin1, TGFR-II, metalloproteinases, epithelial-mesenchymal transition and cell invasion. EGCG also interacts with DNA methyltransferases (DNMTs) and histone deacetylases, restoring ER gene expression, modulating epigenetic changes and interfering with the tumor growth rate [151,152]. Furthermore, studies have demonstrated that EGCG inhibits the growth of TNBC cells through the inactivation of the β-catenin-signaling pathway [153,154]. Remarkably, this antioxidant exhibits anti-migration and pro-apoptosis activities through the activation of caspases 3/7 and upregulation of Bax [155]. Moreover, the combination with curcumin and α-tocopheryl succinate into polystyrene–polysoyaoil–diethanolamine nanoparticles demonstrated to inhibit tumor growth and reduce toxicity when compared with single-drug chemotherapy [156]. Similarly, a combination of EGCG and quercetin, as well as tamoxifen, have an anticarcinogenic effect on both ER-positive and-negative breast cancer cells [157]. Finally, EGCG also possesses chemopreventive potential in breast cancer, suppressing tumor growth through downregulation of the expression of miR-25 and other proteins associated with apoptosis [158]. In summary, as previous studies have shown, EGCG appears to have anticancer activity, and it could be a potential treatment for breast cancer patients. Several registered clinical trials are currently being conducted to test EGCC in breast cancer (Table 9). NCT00917735 was a placebo-controlled, double-blinded, randomized trial from Minnesota, where Samavat et al. investigated the effect of daily supplementation of green tea extract, for one year, on biomarkers of breast cancer risk. They randomized and stratified 1075 healthy postmenopausal women at high risk of breast cancer according to their breast tissue density and catechol-O-methyltransferase genotypes and divided them into two groups: 537 placebo and 538 green tea groups. Green tea group participants took 4 capsules that contained 843 mg EGCG, whereas the placebo group took capsules without green tea extracts. Researchers measured changes in percent mammographic density, circulating endogenous sex hormones, and proteins of the insulin-like growth factor axis [159]. Their results showed that women in the green tea group significantly increased their circulating concentrations of estradiol after supplementation during one year and that the catechol-O-methyltransferase genotype did not influence blood sex hormones before or after supplementation [160]. Moreover, they also suggested that supplementation with green tea extract could modify and reduce mammographic density and protect against breast cancer, even though it was only significant in younger women (50–55 years) and had no effect in older women [161]. Another trial (NCT00516243) regarding green tea and the risk of breast cancer was conducted to determine the maximum tolerated dose of an oral green tea extract, namely polyphenon E (poly-E), a well-defined pharmaceutical-grade decaffeinated green tea catechin mixture that includes epicatechin, epigallocatechin, epicatechin gallate and epigallocatechin gallate. Poly-E (400, 600, 800 mg) was administrated in 30 women twice daily or matching placebo (n = 10 women) for 6 months and determined that the maximum dose was 600 mg twice a day [162]. On the other hand, other trials have focused on the effectiveness of EGCG in treating radiation-induced dermatitis in breast cancer patients undergoing radiotherapy. In one clinical trial, topic EGCG was applied in the initiation of grade I dermatitis for two weeks after termination of the radiotherapy treatment. The majority of patients showed favorable results related to pulling, pain and sensitiveness, suggesting that topic EGCG could be a potential treatment to reduce radiation-induced dermatitis [163,164]. Green tea, like other antioxidant compounds and beverages, has increased in demand; therefore, it is mandatory to examine their safety and toxicity profiles. In vivo assays indicated that topical EGCG preparations caused minor skin irritation in rodents. High oral doses (1000–2000 mg EGCG preparation/kg) were lethal for mice and rats; however, a dose of 200 mg/kg did not induce toxicity. Dietary supplementation of EGCG given to rats for 13 weeks was non-toxic at doses up to 500 mg/kg/day. In humans, there have been no reports of clinical toxicity when green tea is consumed as a drink throughout the day. The administration of EGCG at a daily dose of 800 mg for 4 weeks is safe and well-tolerated in healthy human subjects [165,166,167]. Knowing these data, clinical trials with patients must consider the doses and time of these compounds. Most studies support that EGCG could serve as a promising anticancer agent with clinical applications, but the identification of crucial signaling pathways that interact with EGCG is necessary to improve therapies in breast cancer patients.

### 2.10. Selenium

Selenium is a frequently used antioxidant that has both nutritional and toxicological properties. Exposure to selenium and its supplements has been suggested to protect against various types of cancer, including breast cancer; however, there is controversy over the role of selenium and the risk of breast cancer. Whereas some studies have observed a direct association between this compound concentration in serum and breast cancer risk, suggesting that it could be used as a predictor for breast cancer, other studies do not support this idea [168,169]. Selenium modulates DNA methylation and histone post-translational modifications, and chemical compounds containing selenium, such as methylseleninic acid and selenite, have shown effects on cell proliferation and death in MCF-7 human breast adenocarcinoma cells, reinforcing the anti-breast cancer potential of selenium [170]. Moreover, selenium yeast inhibited the growth of breast cancer cells (MCF-7 and MDA-MB-231) in a dose of 100 to 1500 ng Se/mL, increased oxidative stress and promoted apoptosis without affecting non-tumorigenic cells [171]. Interestingly, while selenium protects the normal tissue from radiation-induced side effects in breast cancer patients, it could also protect tumor cells from radiation-induced cell death, reducing the efficacy of radiotherapy [172]. Concerns about the toxicity of selenium have limited the doses used in chemoprevention. Some cases of selenium toxicity, when consumed in high doses in humans, have been reported to cause selenium poisoning, which induced hair loss or skin lesions [173]. An intake of 400 mg/day, and plasma concentration of 1000 ng/mL, is considered as the level that does not cause adverse effects [174]. A previous study demonstrated that there was a widespread outbreak of selenium toxicity in humans due to a liquid dietary supplement that erroneously contained 200 times of selenium [175]. Future studies may include the analysis and evaluation of selenium species.

Selenium is generally studied in clinical trials to treat side effects of chemotherapy or radiotherapy in breast cancer patients and to improve the quality of life of patients (Table 10). A prospective, randomized, placebo-controlled, double-blind phase-III study was proposed to evaluate whether the application of higher doses of sodium selenite would reduce the toxicity of chemotherapy and radiotherapy [176]. Alternatively, administration of selenite to 48 patients (12 cases of breast cancer) for 4–6 weeks caused a significant reduction of secondarily-developing lymphedema caused by radiation therapy [177]. While in vitro studies show an association of selenium with a decrease in tumor growth, clinical trials have not developed enough evidence suggesting that a higher selenium intake through diet prevents cancer in humans. A retrospective pilot study to evaluate the survival rate in 41 individuals (16 cases of breast cancer), with different end-stage cancer types, who received supplements of coenzyme Q_10_ and a mixture of other antioxidants (vitamin C, selenium, folic acid and β-carotene) (followed-up for >15 years), found that the median survival (in excess over the predicted time) was longer in the 20 patients (9 cases of breast cancer) who began antioxidant treatment within 1.5 months of being diagnosed than in those who began antioxidant treatment later. However, larger clinical trials must be carried out to support the idea that a combination of antioxidants could be used to favor advanced cancer therapies [178]. Additionally, a clinical trial with a double-blinded, placebo control, prospective design in breast cancer patients carrying BRCA1 mutation evaluated whether supplementation with selenium had a beneficial effect in oxidative stress/DNA damage (8-oxodG in cell DNA). It was observed that 8-oxodG levels in the DNA of leukocytes were significantly higher in those cells harboring BRCA1 mutation. In contrast, levels of 8-oxodG in DNA was significantly reduced in the subpopulation of adnexectomized BRCA1 mutation carriers supplemented with selenium in comparison with the subgroup without supplementation. These results suggest that selenium supplementation could decrease oxidative DNA damage and, therefore, a lower risk of developing breast cancer in patients harboring BRCA1 mutation who suffered adnexectomy [179]. Nevertheless, more research is still needed so as to determine whether this compound can ameliorate breast cancer risk in individuals with specific genetic backgrounds or nutritional status.

### 2.11. Synthetic Antioxidants

N-acetyl cysteine (NAC) is a powerful synthetic antioxidant and a precursor of GSH, which reduces oxidative stress and has anticancer properties by reducing proliferation and increasing apoptosis in experimental models with breast cancer cells [180]. Moreover, breast cancer cells treated with NAC have shown attenuated hypoxia-mediated activation of EGFR and less migration capacity under hypoxic conditions [181]. Hereby, tumor exposure to NAC would also enhance trastuzumab-efficacy on HER2-positive breast cancer [182]. Petroleum-derived antioxidants are synthetic compounds that comprise butylated hydroxytoluene (BHT), octyl gallate, butylated hydroxyanisole (BHA), propyl gallate (PG), tert-butylhydroquinone and butylparaben (BuPB) [183]. Interestingly, BHT, together with BHA, PG and BuPB, showed both estrogenic and anti-estrogenic effects in breast cancer [184]. S-BHT, a derivative of BHT, demonstrated a potent antioxidant effect as a free radical scavenger and inhibitory activity against human colon and breast cancer cell lines [185]. Currently, in clinical trials, only NAC is being studied in breast cancer patients, as shown in Table 11. A prospective, randomized, controlled, open-label study assessed the effect of NAC on the incidence and severity of paclitaxel-induced peripheral neuropathy (PIPN) in 75 breast cancer patients for 12 weeks. There were three groups that were administered with oral NAC at a dose of 1200 mg daily, 1200 mg twice daily, and a control group that only received paclitaxel (80 mg/kg). The results suggest that a dose of 1200 mg once and twice a day might reduce the incidence and severity of PIPN and improve the quality of life in breast cancer patients (NCT03492047) [186]. Interestingly, another clinical trial suggested that NAC, as a single agent, could reduce some markers of stromal-cancer metabolic heterogeneity and those related to aggressiveness in human breast cancer. Hence, modulation of the metabolism in the tumor microenvironment could have an impact on breast cancer proliferation. Moreover, the administration of NAC intravenously (150 mg/kg) once a week or orally (600 mg) twice a day reduced MCT4, a marker of glycolysis [187]. On the other hand, as in most other compounds, the toxicity of NAC is rare, and side effects have only been seen when an overdose occurs or when it is administered incorrectly, causing brain dysfunction [188]. Nevertheless, whereas the results from clinical trials are promising, more trials are necessary to improve our understanding of these synthetic antioxidants and their applicability in breast cancer therapies.

## 3. Cancer Stem Cells

One of the main problems facing the fight against cancer is the ability of tumors to cause relapse and metastasis, thus promoting the appearance of new tumors. Cancer stem cells (CSCs), a small subpopulation within the tumor, have the ability to both generate and maintain them [189,190,191,192,193]. CSCs have characteristics of stem cells such as unlimited self-renewal and pluripotency, associated with signaling pathways like Wnt, Notch, and Sonic Hedgehog [194,195], plasticity into the epithelial-to-mesenchymal transition (EMT), which provides them with migratory properties [190,196,197], and the ability to maintain a quiescence state [190,194]. Furthermore, CSCs are crucial in the development of tumors, driving tumorigenesis, drug resistance, treatment resistance, recurrence and metastasis [198,199]. CSCs present multiple independent mechanisms to escape from anticancer drugs, such as upregulation of drug-efflux pumps, a high DNA-repair capacity, or autophagy [200,201]. Moreover, a high expression of ROS-scavenging molecules such as GSH, and enhanced mitochondrial respiratory capacity and high activity of pentose phosphate pathway contribute to their intrinsic resistance to therapies [199,202,203,204]. CSCs can be isolated and characterized by flow cytometry (CD44^+^/CD24^−/low^, ALDH1, or side population, among others) and sphere-forming assays [205]. In the case of breast cancer stem cells (BCSCs), Al-Hajj and colleagues prospectively isolated a tumorigenic population of cells from primary human breast cancer using FACs based on the ESA^+^/CD44^+^/CD24^−/low^/lineage^−^ phenotype [191,193]. Furthermore, by combining ALDH1 (aldehyde dehydrogenase 1) activity with CD44^high^/CD24^−^ expression, the CSC fraction was refined [206,207,208].

In contrast to non-stem like cancer cells, CSCs exhibit a significantly lower level of ROS. CSCs are equipped with an efficient oxidant/antioxidant machinery like peroxiredoxin II, a regulator of ROS in cellular environments by modulation of REDOX status, which helps maintain the stemness and tumorigenic capacities of CSCs [209]. Vitamin C has been proposed as a possible adjuvant, neoadjuvant or cotreatment in the treatment of different types of cancer after showing the capacity to target CSCs when was administered along with chemotherapeutic drugs. More specifically, a combination of vitamin C and doxycycline resulted in a lethal combination therapy targeting the metabolic flexibility in CSCs [28,210]. Additionally, resveratrol and its dimethyl derivative pterostilbene demonstrated to target signaling pathways of CSCs [211]. Similarly, HT proved to reduce BCSC self-renewal, ALDH^+^ and CD44^+^/CD24^−/low^ subpopulations, cell migration and invasion through the alteration of EMT, Wnt/β-catenin and TGFβ-signaling pathways in TNBC cell lines (SUM159, MDA-MB-231 and BT549) [212]. Finally, melatonin seemed to play a pro-autophagic effect in BCSCs derived from MCF-7cells, but an anti-autophagic effect in BCSCs obtained from HEK293 cell [213].

## 4. Immune System, Immunotherapy and Antioxidants in Breast Cancer

Modulation of the immune response by antioxidants has long been proposed as a therapeutic target in cancer, but a direct relationship between them has not been established yet. Antioxidants play an important role in preserving cellular integrity and are critical in maintaining homeostasis of the host’s immune system [214]. Recently, complementary cancer therapies based on antioxidant compounds have been gaining attraction due to their immunomodulating properties and the relevance of immunosuppression in cancer. Immunosuppressive cells within the tumor are determining factors in cancer progression. Thus, the use of antioxidants to attenuate cell proliferation mediated by immunosuppressive cells are extensively studied. Regarding it, resveratrol has been reported to exert an inhibitory effect on these cells. On the one hand, resveratrol, at low doses, has been demonstrated to inhibit lung metastasis in breast cancer by decreasing the generation and function of tumor-evoked regulatory B cells (tBreg) via the inhibition of the STAT3 pathway. As a result, it prevents the tBreg-induced conversion of FoxP3+ Tregs cells, which exert a powerful immunosuppressive character in the tumor microenvironment (TME) and play a crucial role in breast cancer metastasis while remaining non-toxic to effector immune cells [214,215,216]. Moreover, the synthetic resveratrol analog HS-1793 was also shown to decrease the CD4+CD25+ Treg cell population in FM3A breast tumor-bearing mice without affecting the CD4+ T cell population. On the contrary, HS-1793 treatment significantly enhanced tumor-specific cytotoxic T lymphocyte responses in tumor-bearing mice by upregulating the interferon (IFN)-γ-expressing CD8+ T cell population [217,218]. Macrophages, which represent an essential cellular component of the TME in breast cancer, are capable of both promoting and hampering tumor growth and metastasis. Depending on the factors and cytokines of TME, tumor-associated macrophages (TAM) can acquire the M1 or M2 phenotype. M1 polarized macrophages exert antitumor functions, while M2 macrophages have potent immunosuppressive properties, inhibiting the antitumor immune response and promoting tumor progression. TAM has been reported to mostly show the properties of M2 phenotype in breast cancer; thus, they represent a potential target to treat cancer. Concerning this, the increment of IFN-γ production in tumor tissues after HS-1793 treatment was demonstrated to repolarize the immunosuppressive M2 TAM into M1 immunostimulatory macrophages, containing a higher level of proinflammatory and immunostimulatory cytokines [219]. For all those reasons, HS-1793 may serve as a promising adjuvant therapeutic candidate in breast cancer immunotherapy [217,218,219]. Resveratrol also has great potential to evoke an antitumor immune response in patients with breast cancer through the upregulation of protein and mRNA expression of major histocompatibility complex class I chain-related proteins A and B (MICA and MICB), two important ligands for the recognition of tumor cells by natural killer (NK) cells. Thus, resveratrol was suggested to promote the recognition and the subsequent cytolysis of breast cancer cells by NK cells in vitro and in vivo [220]. Moreover, resveratrol was incorporated together with curcumin into immunoliposomes carrying trastuzumab, a HER2-targeted humanized monoclonal antibody approved for clinical use in breast cancer patients, and the properties of these immunoliposomes were studied in two human HER2+ breast cancer cell lines. Immunoliposomes with trastuzumab dramatically enhanced the antitumor therapeutic effect of curcumin and resveratrol in breast cancer cells when compared with free forms. In addition, curcumin increased the cytotoxic effects of the nanovesicles on cancer cells. Hence, these two natural compounds may be potentially administered in immunotherapy protocols [221]. Finally, resveratrol showed a protective effect on the immune system after applying ionizing radiation in mice. In the future, complete well-conducted clinical studies could help elucidate the interplay of resveratrol with the immune system [216].

Other polyphenolic compounds that exert antioxidant and immunomodulatory properties in breast cancer have been examined too. Recent studies have shown that the polyphenol-rich extract (P2Et) modulates the immune system in breast cancer by increasing tumor sensitiveness to chemotherapy, altering the tumor microenvironment and inducing a specific immune response, which reduces metastasis and attacks BCSCs [222,223]. The dietary antioxidant curcumin could partially reverse the inhibition of IL-2 stimulated NK cell tumor cytotoxicity mediated by tumor exosomes, microvesicular bodies containing a distinct set of intracellular and cellular membrane components that are secreted by cancer cells to body fluids. As a consequence, exosomes isolated from breast tumor cells pretreated with curcumin showed a lowered capacity of inhibiting IL-2-stimulated NK cell tumor cytotoxicity [224]. Curcumin may also impair tumor growth through the inhibition of immunosuppressive cells in the TME. Liposomal nanoparticles (NPTs) containing hydrazinocurcumin, a synthetic analog of curcumin, were used to suppress STAT3 activity in M2-like TAMs in a breast cancer model, re-educating TAM to M1 phenotype and restoring the crosstalk with tumor cells. In this way, the re-polarized M1-like macrophages effectively impaired the induction of migration and invasion of 4T1 cells in vitro and inhibited tumor growth, angiogenesis and metastasis in vivo [225,226]. Furthermore, dendrosomal curcumin has been reported to exhibit a similar effect in a murine metastatic breast cancer model, increasing the level of M1-like macrophages while decreasing the level of M2-like macrophages in the TME through the downregulation of STAT3. As a result, tumor volume and weight were significantly reduced after treatment [227]. Due to its immunomodulatory effects, the efficacy of curcumin as an adjuvant in cancer immunotherapies has been evaluated too. An injectable hydrogel containing curcumin-loaded NPTs and a nanovaccine composed of an antigenic peptide and toll-like receptor 9 (TLR) agonist (CpG-ODN) was developed for postoperative tumor immunotherapy. Curcumin NPTs efficiently increased tumor immunogenicity in a model of breast carcinoma through the amplification of the immunogenic cell death (ICD) of tumor cells, which in turn enhanced the antitumor T-cell immunity triggered by the nanovaccine. A single administration of this combination immunotherapy could efficiently attenuate tumor recurrence and lung metastasis of 4T1 tumors [228]. In addition, curcumin has been proved to improve TNBC vaccination with a highly attenuated Listeria monocytogenes (Listeriaat), encoding tumor-associated antigens Mage-b. Curcumin decreased levels of IL-6, a relevant contributor to the immunosuppression in the TME, while increased IL-2 production, which in turn converted myeloid-derived suppressor cells (MDSCs) into an immunostimulating phenotype. Hence, curcumin potentiated antitumor T-cell response and improved the vaccine efficacy of Listeria-Mage-b, achieving a great reduction in the number of metastases in vivo [229]. Similarly, curcumin was proposed to potentially enhance anti-CTLA-4 immunotherapy in breast cancer. This phenolic compound inhibits the COP9 signalosome 5 (CSN5), which is required for tumor necrosis factor-alpha (TNF-α)-mediated PD-L1 stabilization in cancer cells. As curcumin diminished PD-L1 expression in cancer cells, antitumor immunity was improved, and cancer cells were sensitized to anti-CTLA-4 therapy in multiple animal models (including breast cancer), bringing to light the role of curcumin as an adjuvant to immune checkpoint therapies (ICT) [230]. EGCG exerts not only antioxidant and antitumor effects but also immunomodulatory activity [231]. EGCG has been reported to upregulate miR-16 in tumor cells, which may be transported to TAM via tumor-derived exosomes to modulate the immunosuppressive TME. Therefore, EGCG impaired tumor growth in a murine model of breast cancer by both decreasing TAM tumor infiltration and repolarizing M2-like to M1-like macrophages, as evidenced by the diminished IL-6 and TGF-β and increased TNF-α levels [232]. EGCG also inhibited the proliferation of breast cancer cells by significantly reducing the accumulation of MDSCs and increasing tumor infiltration of CD4+ and CD8+ T cells in 4T1 breast tumor-bearing mice [233]. Moreover, EGCG has been used as a layer to encapsulate tumor cells and prepare a vaccine for personalized cancer immunotherapy, potentially customized to individual patient’s tumor cells. Breast cancer cells were successfully coated with the EGCG layer, and this encapsulating strategy enhanced tumor immune responses as well as the clinical efficacy of the cancer vaccine [234].

The potential antitumor and immunomodulatory properties of selenium have been well documented. The combination of an active lifestyle and selenium supplementation could affect antitumor immune responses as well as breast cancer cytokine expression [235]. Regarding it, a nutritional supplement containing selenium and eicosapentaenoic acid (EPA)/docosahexaenoic acid (DHA) was administered to 4T1 tumor-bearing mice together with three chemotherapy agents (Taxol, Adriamycin and Avastin). The combination treatment was proven to enhance the antitumor response of chemotherapy agents through multiple mechanisms (induction of tumor oxidative stress and apoptosis, the inhibition of cancer metastasis and the elimination of BCSCs), resulting in a greater reduction of tumor growth than chemotherapy agents alone. Antitumor Th1-type cytokines were increased, and immunosuppressive Th2-type cytokines were decreased more significantly in vivo after the combination treatment rather than after the individual administration of the chemotherapy agents [236]. The immunostimulatory effect of selenium by the induction of a Th1 immune response in breast cancer was also brought to light in two different studies using selenium nanoparticles as a supplement to treat breast tumor-bearing mice [237,238]. Moreover, different NPTs containing selenium (SeNPs) have been studied to treat breast cancer. Immunotherapy based on immune cell transfer represents one of the most powerful treatment protocols for breast cancer, with a special focus on Vγ9Vδ2T cells, a subset of peripheral γδ T cells with a great antitumor activity that has emerged as a newly discovered promising candidate for solid tumor treatment. Vγ9Vδ2T cells show broader antitumor cytotoxicity than CD4+ and CD8+ T lymphocytes, and they are able to directly target and kill cancer cells via surface receptors (NKG2D) and cytokines (IFN-γ). SeNPs have been shown to potentiate the antitumor activity of γδ T cells in breast cancer through the stabilization of the microtubule network, the upregulation of the expression of cytotoxicity-related molecules (NKG2D, CD16 and IFN-γ) and the downregulation of the immunosuppressive PD-1 expression in these cells. As a result, SeNPs-pretreated Vγ9Vδ2T cells showed stronger cytotoxic activity in vitro and greater tumor growth inhibition efficacy in vivo, when compared with not pretreated Vγ9Vδ2T cells [239]. Another adoptive cell transfer modality that may be enhanced by SeNPs is the cytokine-induced killer cell (CIK)-mediated cancer immunotherapy. As SeNPs upregulate the receptor NKG2D and its ligands expression and activation in CIK, they can be combined with CIK cells to both prolong the in vivo persistence of these cells in peripheral blood and enhance the cytotoxicity of CIK cells from breast cancer patients to tumor cells. Consequently, SeNPs are able to induce tumor infiltration of NK and promote M1 phenotype polarization of TAMs, inhibiting tumor progression in vivo [228]. Selenium was also conjugated with bevacizumab and trastuzumab, and the immunoconjugates have been proposed as a potential combination therapy in the treatment of TNBC cells using various immunotherapeutic approaches that reduce the secondary effects associated with current TNBC anticancer drugs [240].

Other antioxidants, like melatonin, also activate anticancer immune cells, attenuate the immune response of cancer-associated fibroblasts (CAFs) and T regulatory cells, and ameliorate the toxicity induced by ionizing radiation in different organs, therefore enhancing the tumor response to radiotherapy [241,242]. Additionally, ursolic acid carried by liposomes could also effectively reduce tumor CD4+ CD25+ Foxp3+ T cells and MDSCs in 4T1 breast tumor-bearing mice [243]. Furthermore, a novel puerarin nanoemulsion (nanoPue) is able to deactivate stromal CAFs in a TNBC murine model by downregulating ROS production, which is essential to CAFs activation. The activated TME allowed a greater tumor T-cell infiltration, and nanoPue could be successfully synergized with PD-L1 blockade therapy in the TNBC model [233].

Similarly, vitamin C and NAC might be beneficial in the attenuation of immunosuppressive effects of pro-oxidants. Vitamin C accumulates in neutrophils and could contribute to immune defense by improving their movement, phagocytosis, and microbial killing capacities [214,244]. Vitamin C at high-doses has been demonstrated to both delay tumor growth in a T-cell-dependent manner and enhance tumor infiltration of T lymphocytes. Vitamin C could improve tumor cytotoxicity of adoptively transferred CD8+ T lymphocytes and potentiate clinical efficacy of ICT with anti-PD-1 and anti-CTLA-4 monoclonal antibodies in a breast murine model. Therefore, this study brings to light the improvement of the clinical efficacy of immunotherapy by the combination of high doses of vitamin C with ICT [245]. Vitamin C is not the only vitamin that has been reported to have antioxidant and immunomodulatory properties in breast cancer. On the one hand, a maintenance immunotherapy protocol based on low-dose IL-2 and oral 13-cis retinoic acid (RA) has been shown to increase lymphocytes and NK cell levels as well as CD4+/CD8+ ratio in breast cancer patients with a clinical benefit from chemotherapy. RA was combined with IL-2 for its synergistic immunological properties, as RA increases IL-2 receptors and T-helper cell population in peripheral blood. RA is also able to impair tumor-induced T-cell anergy induced by the immature myeloid suppressor cells, improving antitumor immune response in tumor-bearing hosts [246]. On the other hand, α-tocopheryl succinate, the most effective form of vitamin E, which holds great anticancer potential, was delivered by NPTs (NP-TOS15) to 4T1 breast cancer cells, in combination with interferon-gamma (IFN-γ). NP-TOS15 downregulated PD-L1 expression, and the combination therapy enhanced cytotoxic lymphocyte tumor infiltration, inhibiting tumor growth and preventing lung metastasis in 4T1 breast tumor-bearing mice [247]. Additionally, vitamin E was delivered together with paclitaxel into breast cancer cells by a nanoemulsion strategy. Combination therapy exhibited higher cytotoxicity than free paclitaxel in vivo and in vitro, while increased IL-2 secretion and downregulated IL-4 and IL-10 secretion [248]. Moreover, the relationship between vitamin D and the immune system has been analyzed in breast cancer. For instance, calcitriol, the active form of vitamin D, is capable of suppressing miR-320c and miR-520c in breast cancer cells, which in turn upregulated NKG2D ligands MICA/B and unique long 16-binding proteins 1 through 6 (ULBP2) in cancer cells. As a result, calcitriol increased tumor cell sensitivity to NK-mediated cytotoxicity and diminished tumor cell viability [249]. A different mechanism of calcitriol to inhibit the proliferation of TNBC cells consists in the induction of endogenous synthesis of the proinflammatory cytokines IL-1β and TNF-α, which exert their functions through IL-1β receptor 1 (IL-1R1) and TNF-α receptor type 1 (TNFR1), respectively [250]. Vitamin D may alternatively decrease breast tumor growth by augmenting tumor infiltration of CD8+ T cells, with a more active phenotype (T_EM/CM_). However, the effect of this compound depends on diet conditions; in high-fat diet conditions, an opposite effect of vitamin D on breast tumor growth caused by a reduction of CD8+ T cells tumor infiltration was observed [251]. Besides the antiproliferative effect of vitamin D, it might enhance ICT for cancer. Since calcitriol and vitamin D receptors are crucial for the proper activation and effector function of immune cells, particularly T-cells, calcitriol might increase the efficacy of immune checkpoint blockade to promote T-cell activation in order to establish antitumor immunity [252].

Several other compounds with antioxidant activity have been demonstrated to exert an immunomodulatory effect on breast cancer, although they have not been evaluated as adjuvants in any immunotherapy strategy yet. Naringenin could block the secretion of TGF-β1 from breast cancer cells, reducing the percentage of CD4+ CD25+ Foxp3 + T cells and suppressing tumor cell migration as well as lung metastasis [253]. Silibinin reduced MDSCs and increased tumor infiltration of T lymphocytes in 4T1 breast tumor-bearing mice, diminishing tumor volume and enhancing overall survival in the treated mice [254]. Dietary quercetin in combination with intratumor doxorubicin injection was observed to induce a dramatic rejection of 4T1 breast cancer and prevent tumor recurrence in this established breast cancer mouse model. Quercetin induced lymphocyte proliferation and regulated Th1/Th2 balance towards a predominantly Th1 immune response, while the combination therapy promoted a more persistent antitumor immune response [255].

One different approach to treat breast cancer consists of targeting intracellular antioxidant systems present in tumor cells to modulate ROS levels in the TME and improve antitumor immune response. To this end, innovative maleimide liposomes have been developed to deplete intracellular glutathione (GSH) and augment ROS in both breast cancer and dendritic cells (DC). ROS enhances photothermal cancer therapy and induces ICD of tumor cells and promotes DC maturation and tumor antigen presentation. Therefore, this adjuvant therapy increased tumor infiltration of CD8+ T cells, elicited a strong abscopal effect, and significantly prevented tumor metastasis in a murine breast cancer model [256]. Similarly, thioredoxin has been recently evaluated to become a target to deal with TNBC, as this system is upregulated in TNBC cells to maintain homeostasis and is correlated with adverse survival outcomes. Treatment with auranofin, a thioredoxin reductase inhibitor, has been shown to cause specific tumor cell death and hamper the growth of TNBC cells in vitro and in vivo by the generation of high levels of ROS. Furthermore, auranofin exerted a significant antitumor immune response in multiple TNBC models and upregulated PD-L1 expression. Consequently, auranofin was combined with an anti-PD-L1 antibody and the growth of 4T1.2 primary tumors was synergistically impaired [257]. Given the relevance of auranofin and vitamin C as adjuvants with ICT, besides the proven effectiveness of the combined therapy of auranofin and vitamin C against TNBC [104], we hypothesize that ICT could be benefited in combination with both compounds, which warrants further studies and advances.

Considering the broad-spectrum of immunomodulatory functions presented by antioxidant compounds and their clinical potential in breast cancer treatment, it can be concluded that antioxidants may assist cancer therapies to dramatically improve outcomes in aggressive cancers with high mortality rates. Despite further studies in this field are required, preliminary results from studies using antioxidants as a cancer therapy are really encouraging and combining them with current immunotherapies will receive increased interest.

## 5. Lessons from Clinical Trials: Achievements, Challenges and Future Perspectives

It has been demonstrated that antioxidants may present inverse effects on breast cancer; whereas some antioxidants exhibit favorable effects on cancer treatment, others have demonstrated to facilitate tumor initiation and maintenance. Breast cancer is a heterogeneous disease in which different breast cancer subtypes might present different REDOX status. Hence, in order to really understand the potential role of antioxidants in cancer therapy and prevention, we suggest that it would be important to improve the characterization of REDOX status in each subtype, patient and cell type (e.g., TNBC versus ER+, normal weight versus overweight/obese patients, or CSCs versus non-CSCs). Opportunely, we suggest that it would be appropriate to define the concept “redoxidomics” as the discipline to understand the REDOX status, function and interrelationship of the different components of the balance (e.g., enzymes, oxidative stress, antioxidants) and their interaction with other molecules (e.g., DNA, proteins, lipids) that regulate cell fate in an individual. It stems from the integration of oxidomics (as the discipline to understand complex oxidation processes), the REDOX balance, and omics (genomics, proteomics, metabolomics…).

Important achievements in the clinical trials reviewed herein (published in internationally indexed journals in the English language) have been made to help elucidate the role of antioxidants in the management of breast cancer. For instance, melatonin could lead to cancer regression by increasing the efficacy of chemotherapy and reestablish breast tumor sensitivity to this treatment, counteract adverse effects due to chemotherapy (e.g., cognitive function, sleep quality and depressive symptoms). Clinical trials with resveratrol support the chemopreventive effects of its supplementation by modulating estrogen metabolism. In combination with chemotherapy or radiotherapy, curcumin also reduced their side effects such as dermatitis and pain, as well as the important progress that has been made to determine the dose-limiting toxicity (DLT) of curcumin in combination with chemotherapeutics, which has demonstrated effectiveness to decrease angiogenesis. Noteworthy achievements have been done with regards to antioxidant vitamins such as vitamin E as breast cancer chemopreventive, as well as a good inhibitor of radiotherapy-induced fibrosis. Vitamin C improves the immune system, may reduce the risk of breast cancer patient mortality after diagnosis, and counteract the side effects of chemotherapy by the restoration of the antioxidant status of breast cancer patients. Great advances have been achieved on the pro-oxidant role of high doses of vitamin C, as well as a modulator of the tumor cell’s antioxidant response, as adjuvant treatment for breast cancer. The investigation on the role of vitamin D has experienced recent advances that show lower advanced and/or mortality rates among the normal-weight population, which demonstrates the importance of patient stratification according to weight and therefore overcome the volumetric dilution of the compounds administered. Noteworthy, it is known that calcitriol enhances the thioredoxin reductase 1, which is a system involved in the antioxidant status and resistance of tumor-infiltrating NK cells [258]. This relationship should ensure future investigations to determine the potential role of calcitriol on adoptive cell immunotherapy. Vitamin D has also shown the potential to ameliorate musculoskeletal symptoms and arthralgia in breast cancer upon treatment with aromatase inhibitors. Other antioxidants such as carotenoids were correlated with lower breast cancer risk and recurrence in breast cancer survivors, or hydroxytyrosol, which has demonstrated a high potential to improve breast cancer treatments in combination with chemotherapy, as well as to diminish its associated side effects. Finally, epigallocatechin gallate was found to reduce mammographic density, protect against breast cancer and radiation-induced side effects, and selenium, which has been reported as a protector against treatment-related side effects, but little is known about its potential as an adjuvant in breast cancer treatment.

The use of antioxidants, either phytochemical or synthetic, continues to be controversial because of the difficulty to determine whether their supplementation can positively affect therapeutic outcomes or improve adverse effects induced by chemotherapy, radiotherapy or even immunotherapy. Be that as it may, the insufficient number of clinical studies and some experimental flaws not addressed by them makes it difficult to ascertain whether antioxidants may be potential therapeutic adjuvants, which is the main topic of this review. In line with other authors [22,25,259,260] we highlight common breaches in clinical trials that challenge a successful translation of antioxidants into cancer management, such as: route of administration; difficulty to achieve maintained therapeutic concentrations; low patient number; lack of previous preclinical assessment of a safe, effective and clinically relevant dose regimen for a given antioxidant (as monotherapy and combined with other therapeutics such as chemotherapy, radiotherapy or immunotherapy) in appropriate models of the breast cancer subtype under investigation (preferably patient-derived xenografts and/or organoids); non-standardized readouts (such as the impact on patient’s “redoxidomics”, gene and/or protein expression, post-translational protein modifications at tumor and antioxidant defense levels, or even on immune system that includes tumor-infiltrating lymphocytes or tumor-associated macrophages) and patient stratification (adjuvant or neoadjuvant therapy, “redoxidomic” status, breast tumor subtype, nutritional state, bodyweight or healthy habits followed by case subjects); lack of information about the impact on clinical outcome (overall, relapse-and metastasis-free survival); and low multidisciplinary teams.

**Figure 1:**
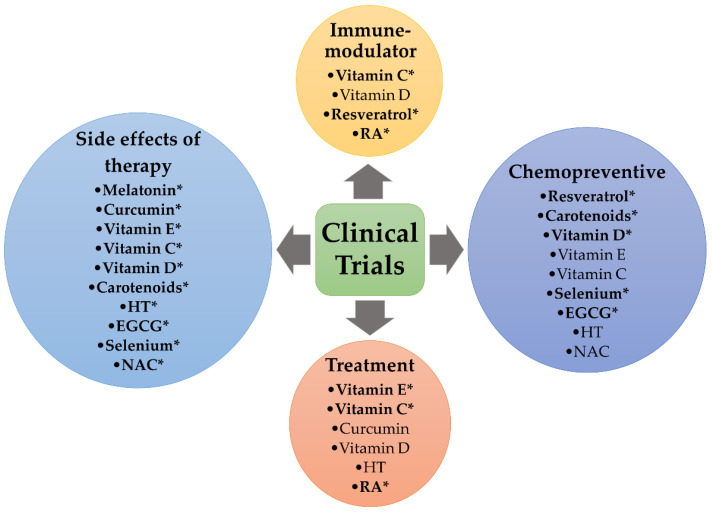
Antioxidants studied in clinical trials in breast cancer patients based on their properties as chemopreventives, adjuvants in treatments, and modulators of the immune system and therapy-derived side effects. Asterisk (*) indicates that a given property has been confirmed in clinical studies. Epigallocatechin gallate (EGCG); hydroxytyrosol (HT); N-acetyl cysteine (NAC); retinoic acid (RA).

Although there is growing interest in how antioxidants may influence breast cancer treatments, traditionally, most clinical trials have focused on the amelioration of the therapy-derived side effects and on their supplementation as chemopreventive agents in breast cancer (Figure 1). Factually, supplementation could be one of the main mistakes that scientists may be making in their clinical investigations. The fact is that oral supplementation of antioxidants could be appropriate when chemoprevention or alleviation of treatment-derived side effects is the goal of a study. However, when antioxidants are intended as therapeutic adjuvants, doses must be higher and long-lasting, and, therefore, the administration route should be carefully established. In this sense, oral supplementation may reduce the bioavailability of bioactive forms of antioxidants and their plasmatic concentrations, whereas whether intravenous injection will allow achieving sustained high concentrations of the bioactive forms remains unknown, and it warrants further investigations.

## 6. Conclusions

In conclusion, more research at the preclinical level (both in vitro and in vivo) must be done in order to clarify the role of antioxidants in cancer initiation and progression, whether they have a dual role (antioxidant or oxidant) depending on the concentration, and how these compounds and therapies interact. In fact, doses and/or actual bioavailability of the supplementary anticancer therapy seems to play a crucial part, whether a phytochemical is potentially an antioxidant or oxidant agent, thus justifying further personalized clinical/genetic studies. Notwithstanding, it is mandatory to redefine important aspects in clinical trials, being crucial the route of administration, patient stratification according to their oxidative stress status (expression of antioxidant/oxidant enzymes, antioxidant capacity, the grade of DNA damage, among others, or “redoxidomics”, systemically and in tumor tissue), breast tumor subtype, and body weight to better determine the correct dose and timing of antioxidant supplementation for future standardized and personalized treatments of patients. Undoubtedly, for over 50 years, human beings have taken advantage of natural products (plant, microbial or marine origin) as anti-breast cancer therapeutics (e.g., taxanes, vinca alkaloids vinblastine, vincristine, camptothecin, etoposide, teniposide, anthracyclines, epothilones, trabectedin, among others) [261]. Hence, in remembrance of those early advances that led to a new era in the treatment of cancer, and to the best of our knowledge, we encourage clinical and translational scientists to embrace the possibility that some antioxidant compounds deserve the opportunity to become part of such a venerable group of therapeutics, beyond being mere chemopreventive agents.

Finally, in answer to the question addressed at the onset of this review, “Antioxidants for the treatment of breast cancer: are we there yet?”, besides the necessity of further investigations, we conclude that the most promising results from clinical trials have been obtained for vitamin C and its combination with vitamin E, therefore, these compounds are close to being a reality in breast cancer therapy.

## Figures and Tables

**Table 1:** Clinical trials with melatonin in breast cancer.

NCT Number	Status ^1^	Start/Completion Date ^1^	Stage	Title	References
NCT03205033	Completed	01/2016–01/2017	Phase II	Melatonin as a circadian clock regulator, neuromodulator and myelo-protector in adjuvant breast cancer chemotherapy	[41,53]
NCT01355523	Terminated	07/2011–01/2013	Phase II/III	The effect of melatonin on depression, anxiety, cognitive function and sleep disturbances in breast cancer patients (MELODY)	[49,50]
NCT01805089	Completed	10/2006–07/2009	Early phase I	Melatonin versus placebo in breast cancer	NP
NCT01557478	Unknown	03/2012–present	Phase II/III	Melatonin as adjuvant therapy in breast cancer patients (MIQOL-B)	NP
NCT01965522	Completed	10/2013–05/2017	Phase II	Antiproliferative effects of vitamin d and melatonin in breast cancer (MELO-D)	NP
NCT02506777	Recruiting	07/2015–present	Phase II	Neoadjuvant FDC with melatonin or metformin for locally advanced breast cancer (MBC1)	NP
NCT00506064	Terminated due to low accrual	02/2004–09/2008	Phase I	Melatonin postoperative sleep study in breast cancer patients	NP
NCT02506790	Recruiting	07/2015–present	Phase II	Neoadjuvant toremifene with melatonin or metformin in locally advanced breast cancer	NP
NCT03716583	Recruiting	04/2019–present	Phase II	Melatonin cream against acute radiation dermatitis in patients with early breast cancer (MELADERM)	NP
NCT02332928	Recruiting	03/2015–present	Phase III	Melatonin supplementation for cancer-related fatigue in patients receiving radiotherapy	NP
NCT01171508	Completed	02/2011–11/2011	Not applicable	Circadian disturbances after breast cancer surgery (CIRCA)	NP
NCT02486796	Terminated	02/2016–03/2017	Phase I/II	Immediate or delayed naturopathic medicine in combination with neoadjuvant chemotherapy for breast cancer	NP
NCT00519168	Completed	09/2006–08/2011	Not applicable	Sleep, circadian hormonal dysregulation, and breast cancer survival	NP
NCT02883790	Terminated	10/2015–02/2018	Not applicable	Effects of Somnage^®^ in the management on sleep and mood in cancer patients	NP
NCT03511079	Recruiting	07/2019–present	Not applicable	Music as a perioperative therapy in breast cancer patients	NP
NCT04418856	Recruiting	06/2020–present	Not applicable	The effects of light therapy to treat cancer-related side effects	NP
NCT04364347	Recruiting	12/2019–present	Not applicable	Chemotherapy-induced circadian rhythm disruption	NP
NCT04401189	Not yet recruiting	06/2020–present	Not applicable	The role of circadian rhythms in cancer-related symptoms (CHRONO)	NP
NCT02011815	Completed	11/2013–06/2019	Not applicable	Exploring the biological linkage between circadian disruption and cancer progression	NP
NCT02609373	Completed	07/2011–01/2016	Not applicable	Improving cancer-related outcomes in shift workers (ICOS)	NP

^1^ Last access to ClinicalTrials.gov on 14 December 2020. NP—not published.

**Table 2:** Clinical trials with resveratrol in breast cancer.

NCT Number	Status ^1^	Start/Completion Date ^1^	Stage	Title	References
NCT01370889	Completed	06/2011–07/2012	Phase I	Resveratrol in postmenopausal women with high body mass index	[66]
NCT03482401	Completed	06/2017–31/2019	Not applicable	Disposition of dietary polyphenols and methylxanthine in mammary tissues from breast cancer patients (POLYSEN)	[67]
NCT04266353	Suspended due to COVID-19	04/2019–08/2020	Not applicable	Effect of resveratrol on serum IGF2 among African American women	NP

^1^ Last access to ClinicalTrials.gov on 14 December 2020. NP—not published.

**Table 3:** Clinical trials with curcumin in breast cancer.

NCT Number	Status ^1^	Start/Completion Date ^1^	Stage	Title	References
NCT01042938	Completed	01/2008–04/2011	Phase II	Curcumin for the prevention of radiation-induced dermatitis in breast cancer patients	[84]
NCT02556632	Completed	10/2015–09/2016	Phase II	Prophylactic topical agents in reducing radiation-induced dermatitis in patients with non-inflammatory breast cancer (curcumin-II)	[85]
NCT03980509	Recruiting	06/2020–present	Phase I	A window trial on curcumin for invasive breast cancer primary tumors	NP
NCT03847623	Active, not recruiting	06/2017–present	Not applicable	Effect of preoperative curcumin in breast cancer patients (EPC)	NP
NCT03865992	Recruiting	03/2019–present	Phase II	Curcumin in reducing joint pain in breast cancer survivors with aromatase inhibitor-induced joint disease	NP
NCT03072992	Completed	03/2017–06/2019	Not applicable	Curcumin in combination with chemotherapy in advanced breast cancer	NP
NCT01740323	Completed	05/2015–07/2018	Phase II	Phase II study of curcumin vs. placebo for chemotherapy-treated breast cancer patients undergoing radiotherapy	NP
NCT01975363	Completed	06/2013–09/2016	Not applicable	Pilot study of curcumin for women with obesity and high-risk for breast cancer	NP
NCT00852332	Terminated	08/2009–11/2017	Phase II	Docetaxel with or without a phytochemical in treating patients with breast cancer	NP
NCT01246973	Completed	02/2011–01/2015	Phase II/III	Oral curcumin for radiation dermatitis	NP
NCT03482401	Completed	06/2017–12/2019	Not applicable	Disposition of dietary polyphenols and methylxanthine in mammary tissues from breast cancer patients (POLYSEN)	NP

^1^ Last access to ClinicalTrials.gov on 14 December 2020. NP—Not published.

**Table 4:** Clinical trials with vitamin E in breast cancer.

NCT Number	Status ^1^	Start/Completion Date ^1^	Stage	Title	References
NCT00583700	Completed	02/2003–06/2012	Phase II	Trental and vitamin E for radiation-induced fibrosis	[99]
NCT01157026	Completed	11/2001–01/2010	Not applicable	A pilot clinical trial with tocotrienol on breast cancer	[100]
NCT04463459	Recruiting	10/2019–present	Not applicable	Effect of vitamin C and E in breast cancer patients undergoing chemotherapy	NP
NCT03916068	Recruiting	07/2019–present	Phase II	Acute post-radiation hyperbaric oxygen (HBO2) for breast cancer patients who have recently completed radiation therapy	NP
NCT04496492	Completed	02/2016–07/2017	Phase II	Preoperative use of tocotrienol from *Annatto bixa orellana l.* in breast cancer patients: a prospective clinical trial	NP
NCT03855423	Recruiting	02/2019–present	Not applicable	Maximum tolerated dose, safety and pharmacologic study of TRF in women with breast cancer (Matriac)	NP
NCT02909751	Active, not recruiting	09/2016–present	Phase II	Tocotrienol in combination with neoadjuvant chemotherapy for women with breast cancer (NeoToc)	NP
NCT00022204	Completed	01/2000–unknown	Phase II	Vitamin E and pentoxifylline in treating women with lymphedema after radiation therapy for breast cancer	NP
NCT02898376	Not yet recruiting	12/2018–present	Phase III	Clinical benefit of spa care on severe radiation-induced fibrosis after postoperative radiotherapy for breast cancer (FIBROTHERME)	NP
NCT00188669	Terminated	07/2002–unknown	Phase II	The use of pentoxifylline and vitamin E in the treatment of chronic breast pain	NP
NCT01571921	Completed	01/2013–02/2013	Phase I	Gamma-delta tocotrienol as potential maintenance treatment in women with metastatic breast cancer (GEMM1a)	NP
NCT04446624	Completed	02/2018–12/2019	Not applicable	Oxidative stress, anxiety and depression in breast cancer patients: impact of music therapy	NP

^1^ Last access to ClinicalTrials.gov on 14 December 2020. NP—not published.

**Table 5:** Clinical trials with vitamin C in breast cancer.

NCT Number	Status ^1^	Start/Completion Date ^1^	Stage	Title	References
NCT04463459	Recruiting	10/2019–present	Not applicable	Effect of vitamin C and E in breast cancer patients undergoing chemotherapy	NP
NCT03175341	Unknown	10/2018–present	Phase I/II	Intravenous ascorbic acid supplementation in neoadjuvant chemotherapy for breast cancer	NP
NCT02521077	Withdrawn	Not applicable	Phase II	Intravenous ascorbic acid in women receiving adjuvant or neoadjuvant chemotherapy for early-stage breast cancer	NP

^1^ Last access to ClinicalTrials.gov on 14 December 2020. NP—not published.

**Table 6:** Clinical trials with vitamin D in breast cancer.

NCT Number	Status ^1^	Start/Completion Date ^1^	Stage	Title	References
NCT01169259	Active, not recruiting	07/2012–present	Phase III	Vitamin D and omega-3 trial (VITAL)	[118]
NCT00003787	Completed	03/1995–12/2018	Not applicable	Women’s healthy eating and living study	[121,122]
NCT01948128	Completed	10/2013–09/2015	Phase II	Effects of vitamin D in patients with breast cancer (OTT 12-06)	[123]
NCT00867217	Completed	03/2009–01/2011	Phase II	Vitamin D3 for aromatase inhibitor-induced arthralgias (VITAL)	[128]
NCT01224678	Completed	10/2010–12/2014	Phase III	Vitamin D and breast cancer biomarkers in female patients	NP
NCT01166763	Completed	05/2009–06/2011	Not applicable	Modulation of breast cancer risk biomarkers by high dose vitamin D	NP
NCT04091178	Completed	10/2013–03/2017	Phase II	Vitamin D supplementation to correct the vitamin D deficiency for breast cancer (OPTIVIT)	NP
NCT00976339	Completed	09/2007–12/2013	Phase I	Study of vitamin D for premenopausal women at high risk for breast cancer	NP
NCT01472445	Terminated	11/2011–10/2015	Phase II	Vitamin D and breast cancer: does weight make a difference?	NP
NCT01480869	Completed	07/2011–12/2014	Phase III	Study of vitamin D supplementation tailored to vitamin D deficiency in breast cancer patients (VITACAL)	NP
NCT00656019	Completed	04/2018–12/2011	Phase II	Development of vitamin D as a therapy for breast cancer	NP
NCT00859651	Completed	06/2009–04/2015	Phase II	Vitamin D in postmenopausal women at high risk for breast cancer	NP
NCT01817231	Completed	05/2009–08/2009	Not applicable	Epidemiological analysis of vitamin D and breast cancer risk in Saudi Arabian women	NP
NCT04166253	Recruiting	01/2020–present	Phase II	Protective role of vitamin D in breast cancer patients treated with doxorubicin (VDDOXO)	NP
NCT01965522	Completed	10/2013–05/2017	Phase II	Antiproliferative effects of vitamin d and melatonin in breast cancer (MELO-D)	NP
NCT00944424	Unknown	07/2009–present	Phase III	Phase III trial of high dose vs. standard-dose vitamin D2 with docetaxel in metastatic breast cancer (GORG-002)	NP
NCT01988090	Terminated	12/2013–12/2018	Phase II	High-dose vitamin D vs. standard-dose vitamin D study	NP
NCT02786875	Recruiting	11/2016–present	Phase III	Diet, exercise and vitamin D in breast cancer recurrence (DEDiCa)	NP
NCT01809171	Terminated	10/2013–08/2015	Phase II	Placebo-controlled trial with vitamin D to prevent worsening/relieve aromatase inhibitor-induced musculoskeletal symptoms in breast cancer patients	NP
NCT02856503	Withdrawn	Not applicable	Phase I/II	Effect of high dose vitamin D on cancer biomarkers and breast cancer tumors	NP
NCT00263185	Completed	11/2005–11/2009	Phase I	High-dose vitamin D musculoskeletal symptoms and bone density in anastrozole-treated breast cancer with marginal vitamin D status	NP
NCT02186015	Completed	02/2015–11/2017	Phase II	Safety, feasibility and efficacy of vitamin D supplementation in women with metastatic breast cancer (SAFE-D)	NP
NCT01608451	Active, not recruiting	09/2007–present	Phase III	Randomized controlled trial of neo-adjuvant progesterone and vitamin D3 in women with large operable breast cancer and locally advanced breast cancer	NP
NCT01425476	Completed	07/2008–11/2016	Phase I/II	Changes in breast cancer biomarkers using synergistic prostaglandin inhibitors	NP
NCT00022087	Completed	12/2011–02/2009	Phase III	Zoledronate, calcium, and vitamin D in preventing bone loss in women receiving adjuvant chemotherapy for breast cancer	NP
NCT01816555	Terminated	01/2013–11/2014	Phase I	Vitamin D3 (Vit D3) supplementation and t cell immunomodulation in patients with newly diagnosed operative invasive ductal breast carcinoma	NP
NCT01747720	Completed	10/2012–05/2017	Not applicable	Vitamin D and mammographic breast density (EVIDENSE)	NP
NCT00904423	Terminated	04/2009–04/2011	Phase I/II	Ph I/II of vitamin D on bone mineral density and markers of bone resorption	NP
NCT03594214	Not yet recruiting	09/2018–present	Not applicable	Prognostic value of vitamin D levels in Egyptian females with breast cancer	NP
NCT01240213	Completed	10/2010–09/2012	Not applicable	Vitamin D, diet and activity study (ViDA)	NP
NCT03986268	Recruiting	05/2019–present	Not applicable	Vitamin D can increase the pathological response of the breast cancer patients treated with neoadjuvant therapy	NP
NCT01769625	Completed	01/2009–11/2016	Phase I/II	Changes in biomarkers using prostaglandin inhibitors	NP
NCT00926315	Unknown	06/2007–present	Not applicable	Gene expression profile of breast cancer samples after vitamin D supplementation	NP
NCT02936999	Terminated	08/2016–01/2019	Not applicable	Vitamin D supplementation in women with DCIS and/or LCIS	NP
NCT00054418	Completed	03/2003–05/2008	Phase III	Risedronate in preventing bone loss in premenopausal women receiving chemotherapy for primary breast cancer	NP
NCT00416715	Completed	10/2006–05/2010	Phase II	Vitamin D deficiency, muscle pain, joint pain, and joint stiffness in postmenopausal women receiving letrozole for stage I-III breast cancer	NP
NCT01097278	Completed	11/2011–09/2017	Not applicable	S0812 high dose cholecalciferol in premenopausal women at high-risk for breast cancer	NP
NCT01419730	Completed	08/2011–05/2020	Phase II	Vitamin D and physical activity on bone health	NP
NCT00567606	Completed	04/2002–12/2007	Phase IV	Prevention of osteoporosis in breast cancer survivors	NP

^1^ Last access to ClinicalTrials.gov on 14 December 2020. NP—not published.

**Table 7:** Clinical trials with carotenoids in breast cancer.

NCT Number	Status ^1^	Start/Completion Date ^1^	Stage	Title	References
NCT00000611	Completed	10/1999–04/2016	Phase III	Women’s Health Initiative (WHI)	[137]
NCT03625635	Unknown	09/2015–08/2019	Not applicable	Effect of a clinical nutrition intervention program in breast cancer patients during antineoplastic treatment	NP
NCT04374747	Recruiting	10/2019–present	Not applicable	Fruit and vegetable intervention in lactating women to reduce breast cancer risk	NP
NCT02109068	Completed	01/2011–01/2014	Phase III	Lifestyle, exercise and nutrition study 1 (LEAN)	NP
NCT02110641	Active, no recruiting	11/2013–present	Not applicable	Lifestyle, exercise and nutrition study 2 (LEAN 2)	NP
NCT02067481	Completed	03/2013–07/2013	Phase II	Effect of a diet and physical activity intervention in breast cancer survivors (PREDICOP-F)	NP
NCT04446624	Completed	02/2018–12/2019	Not applicable	Oxidative stress, anxiety and depression in breast cancer patients: impact of music therapy	NP

^1^ Last access to ClinicalTrials.gov on 14 December 2020. NP—not published.

**Table 8:** Clinical trials with hydroxytyrosol in breast cancer.

NCT Number	Status ^1^	Start/Completion Date ^1^	Stage	Title	Reference
NCT01819948	Completed	06/2012–12/2015	Not applicable	Changes in biomarkers of cancer in women with breast cancer and without evidence of disease who were given PhytoMed™	[150]
NCT02068092	Recruiting	12/2013–present	Phase II/III	Olive oil for the prevention in women at high risk of breast cancer	NP
NCT03482401	Completed	06/2017–12/2019	Not applicable	Disposition of dietary polyphenols and methylxanthine in mammary tissues from breast cancer patients (POLYSEN)	NP

^1^ Last access to ClinicalTrials.gov on 14 December 2020. NP—not published.

**Table 9:** Clinical trials with epigallocatechin gallate (EGCG) in breast cancer.

NCT Number	Status ^1^	Start/Completion Date ^1^	Stage	Title	References
NCT00917735	Completed	07/2009–06/2014	Phase II	Green tea and reduction of breast cancer risk	[159,160,161]
NCT00516243	Completed	07/2007–03/2010	Phase I	Defined green tea catechin extract in treating women with hormone receptor-negative stage I-III breast cancer	[162]
NCT01481818	Enrolling by invitation	09/2011–present	Phase I/II	Study of topically applied green tea extract for radiodermatitis and radiation mucositis	[163,164]
NCT00949923	Completed	05/2008–06/2016	Not applicable	Green tea in breast cancer patients	NP
NCT02580279	Enrolling by invitation	12/2014–10/2019	Phase II	Study of epigallocatechin-3-gallate (EGCG) for skin prevention in patients with breast cancer receiving adjuvant radiotherapy	NP

^1^ Last access to ClinicalTrials.gov on 14 December 2020. NP—not published.

**Table 10:** Clinical trials with selenium in breast cancer.

NCT Number	Status ^1^	Start/Completion Date ^1^	Stage	Title	References
NCT00555386	Completed	04/2007–08/2008	Not applicable	Soy, selenium and breast cancer risk	NP
NCT00188604	Completed	01/2004–01/2009	Phase II	The use of selenium to treat secondary lymphedema-breast cancer	NP
NCT04014283	Recruiting	10/2014–present	Not applicable	Prevention of female cancers by optimization of selenium levels in the organism (SELINA)	NP
NCT01611038	Completed	10/2011–06/2015	Not applicable	Methyl selenocysteine effects on circadian rhythm	NP
NCT00160901	Completed	08/2003–12/2005	Phase IV	Complementary therapies for the reduction of side effects during chemotherapy for breast cancer	NP

^1^ Last access to ClinicalTrials.gov on 14 December 2020. NP—not published.

**Table 11:** Clinical trials with synthetic antioxidants in breast cancer.

NCT Number	Status ^1^	Start/Completion Date ^1^	Stage	Title	References
NCT03492047	Completed	04/2018–06/2019	Phase I/II	N-acetyl cysteine effect in peripheral neuropathy in cancer patients	[186]
NCT01878695	Completed	07/2012–05/2015	Phase I	Pilot study of antioxidant supplementation with N-acetyl cysteine in stage 0/I breast cancer (NAC)	[187]

^1^ Last access to ClinicalTrials.gov on 14 December 2020. NP—not published.

## Abbreviations

1,25(OH)_2_D_3_	1α,25-dihydroxyvitamin D_3_
25(OH)D_3_	25-hydroxyvitamin D_3_
BCSCs	Breast cancer stem cells
BHT	Butylated hydroxytoluene
BuPB	Butylparaben
CAFs	Cancer-associated fibroblasts
CAT	Catalase
CIK	Cytokine-induced killer cell
CSCs	Cancer stem cells
DC	Dendritic cells
DHEA	Steroid dehydroepiandrosterone
DLT	Dose-limiting toxicity
EGCG	Epigallocatechin gallate
ER	Estrogen receptor
GSH	Glutathione
HER2	Human epidermal growth factor receptor 2
HPIMBD	4-(E)-{(4-hydroxyphenylimino)-methylbenzene-1,2-diol}
HT	Hydroxytyrosol
ICD	Immunogenic cell death
ICT	Immune checkpoint therapies
IGF-1	Insulin-like growth factor I
MDSCs	Myeloid-derived suppressor cells
NAC	N-acetylcysteine
NP	Not published
NPTs	Nanoparticles
PG	Propyl gallate
PIPN	Paclitaxel-induced peripheral neuropathy
PTX	Pentoxifylline
RA	Retinoic acid
RDS	Radiation dermatitis severity
ROCK-1	Rho-associated kinase protein
SOD	Superoxide dismutase
STAT3	Signal transducer and activator of transcription 3
tBreg	Tumor-evoked regulatory b cells
TAM	Tumor-associated macrophages
TIMBD	4-(E)-{(p-tolylimino)-methylbenzene-1,2-diol} ()
TME	Tumor microenvironment
TNBC	Triple-negative breast cancer
TRF	Tocotrienol rich fraction
